# Robust mask-based appointment scheduling in primary care practices

**DOI:** 10.1007/s10729-026-09789-z

**Published:** 2026-10-31

**Authors:** Sabrina Schmitz, Martin Comis, Christina Büsing

**Affiliations:** Combinatorial Optimization, RWTH Aachen University, Im Süsterfeld 9, 52072 Aachen, Germany

**Keywords:** Primary health care, Appointment scheduling, Demand uncertainty, Robust minimum cost flows

## Abstract

In most health care systems, a primary care physician (PCP) is both the first instance consulted by patients with medical concerns and the instance coordinating patients’ continued access to medical care. Due to the PCP’s pivotal role, we address challenges of a high-quality primary care service by interday appointment scheduling on a tactical decision level. In addition to patients who seek medical care by appointments, we also consider patients who complicate the PCP’s schedule planning by seeking immediate care as walk-ins without prior notice or failing to show up to their appointments. We study appointment scheduling systems based on so-called masks and focus on the balanced workload of the PCP in form of the mask design problem. To account for different uncertainties in demand for treatment, we extend the mask design problem to the robust mask design and the robust multimask design problem. For all three problems, we provide a combinatorial interpretation by a network flow and design model. We develop a solution approach that combines binary search with compact formulations (of extensions) of minimum cost flow problems. Finally, we conduct an extensive case study by agent-based simulation. We evaluate the mask-based appointment scheduling systems and compare them with five appointment scheduling systems from the literature on the basis of key performance indicators such as the PCP’s overtime and idle time as well as the number of unsuccessful treatment requests or rejected walk-ins.

## Introduction

### Motivation

Primary care physicians (PCPs) play a crucial role in health care systems as they are often the first instance consulted by patients with medical concerns [1, 2]. PCPs’ responsibilities begin with conducting an examination to determine the patients’ states of health and assessing whether they themselves can provide the necessary medical care. If this is not the case, they are obliged to ensure the continuation of medical care, for example by referring the patient to a specialist [3]. As the instance facilitating and coordinating patients’ access to medical care, the PCP’s quality of service is not only measured on the basis of competent medical care but also by aspects such as the patient-physician continuity, timely access to medical care, and waiting times within the practice, as discussed in more detail in the following.

Patient-physician continuity is desirable as patients benefit from follow-up treatments and referrals [3, 4]. PCPs themselves benefit as they have to spend less time dealing with medical histories of new patients [1]. Clearly, patient-physician continuity may conflict with swift access to health care if patients prefer to be treated by their familiar PCP rather than by any available PCP [5]. Timely access to medical care is imperative, as it is decisive for the patients’ choice of medical facility, course of illness, and medical outcome [1, 6]. For instance, long access times for PCP practices increase the likelihood of patients visiting emergency rooms [7, 8]. In the United States, patients’ timely access is endangered by the clash of increasing demand for primary health care and the nationwide lack of PCPs [2, 9]. Similar conflicts arise in other countries. For instance in rural areas of Germany, mainly elderly PCPs near retirement [10] combined with fewer medical graduates who are willing to start a practice [11] threaten the prospect of timely medical care. Due to the worldwide aging population with the highest risk of chronic conditions [9, 12], PCPs’ schedules are already overloaded [13]. Overloading in turn leads to long waiting times in the practice—one of the major reasons for patients’ complaints [14].

To face the described and further challenges with providing high-quality service, an effective appointment scheduling system is required [15, 16]. However, the PCPs’ schedule planning is complicated as patients do not attend their appointments or patients forgo scheduling an appointment and seek immediate care by walking into the practice without prior notice—as so-called no-shows or walk-ins. In the United States, no-show and walk-in rates range between 20% to 30% and 10% to 25% of daily primary care visits, respectively [17, 18]. German surveys reveal similar data regarding walk-ins, namely that an average of 18.8% of patients are walk-ins [19]. Compared to data for specialists with an average rate of 6.5%, this almost three times higher walk-in rate highlights the difficulty of scheduling in German primary care practices. In addition to high numbers of walk-ins and/or no-shows, the scheduling is further complicated by fluctuating demand for treatment which leads to an inefficient and imbalanced workflow of PCPs [20]. PCPs have to work overtime on one day and are underutilized on another day of the same week. In summary, an effective appointment scheduling system maintains patient-physician continuity, enables timely access, reduces waiting times, smooths the PCP workflow, and provides robustness against demand uncertainties [1].

Contributing to the efforts of determining an effective appointment scheduling system, we study the appointment scheduling problem on a tactical decision level in form of a so-called mask design problem (MDP). The MDP addresses the problem of matching the PCP’s capacity and the demand for treatment of different patient types with different needs and behaviors. For this purpose, the PCP works with a so-called mask designed for their appointment schedule that specifies which time slots are intended for which patients. We consider patients who seek medial care by appointment but may not show up to their appointments as well as patients who seek medical care as walk-ins. To account for uncertain demand for treatment, we extend the MDP to the robust mask design problem (rMDP) and the robust multimask design problem (rMMDP). To that end, we model uncertain demands for treatment using potential scenarios that may occur, which we integrate into our model in a robust optimization framework. The main focus of this paper is the rMMDP for which we aim at finding multiple masks that are robust against demand uncertainties and between which PCPs can switch to flexibly react to changes in demand. For instance, if a PCP uses a mask that is tailored to low demand and notices an increase in demand, they can switch to a mask designed for that level of demand. Before discussing previous related work and go into further detail, we stress our contribution to the field.

### Contribution and outline

The main contributions of this paper are summarized as follows. We define the MDP considering patients who seek medical care by appointment (but may not show up) and patients who seek medical care as walk-ins. For this problem, we present a network flow and design model using a solution approach that combines binary search and compact integer program (IP) formulations of minimum cost flow problems. Due to uncertainties in demand for treatments, we extend the MDP to the rMDP. To account for additional uncertainties and overfitted solutions, we present the rMMDP. For the rMDP and rMMDP, we extend our solution approach by robust compact minimum cost flow formulations. To analyze the resulting mask-based appointment scheduling systems, we conduct a case study using agent-based simulation. In this case study, we test on an operational decision level whether the mask-based appointment scheduling systems can withstand a complex primary health care system that includes individual patient events and characteristics such as cancellations, the willingness to wait to an appointment, and session preferences. We evaluate that the robust mask-based appointment scheduling systems lead to high-quality solutions in several key performance indicators. Compared to the appointment scheduling systems from the literature, for example, the number of patients’ unsuccessful appointment requests is halved and the number of rejected walk-ins is reduced to one third. In addition, patients’ access times to appointments is shortened and PCP’s overtime is reduced. To the best of our knowledge, this is the first contribution that studies interday appointment scheduling by means of combinatorial and robust methods. More precisely, this is the first contribution that studies interday appointment scheduling as a robust network flow and design problem.

The paper is structured as follows. In Section 2, we outline literature of appointment scheduling in primary health care with a focus on interday appointment scheduling. In Section 3, we define a physician-patients setting, introduce mask-based interday appointment scheduling, and present the MDP. In Section 4, we model the MDP in a deterministic setting and subsequently extend it to a robust setting. In Section 5, we consider a further extension of the MDP, the rMMDP. We note that the modeling in Sections 4 and 5 is presented step-by-step in great detail. In Section 6, we present and discuss the results from our case study. In Section 7, we discuss and conclude our results.

## Appointment scheduling in the literature

Health care services face many challenges due to the clash of increasing demand for medical care and the resources available [21, 22]. In the overview [23], Hulshof et al. identify the general classifications of planning and control decisions of health care services. Concentrating on appointment scheduling in outpatient services, we refer to the overview of Cayirli and Veral [24] which focuses on formulating general problems and their various modelings. Factors that make appointment scheduling challenging are demonstrated by Gupta and Denton [1]. Furthermore, we refer to Ahmadi-Javid et al. [25] for a review of analytical and numerical optimization studies for outpatient appointment scheduling systems. In the following, we provide an overview about the categories into which appointment scheduling systems are classified as well as related work.

### Categories of appointment scheduling systems

Appointment scheduling systems established in the literature are categorized into four different types of systems [26]: traditional, advanced access, hybrid, and carve-out appointment scheduling systems. The traditional appointment scheduling system allows all slots to be scheduled for appointments by requesting patients. Appointments may be scheduled well in advance and are often used to postpone patients requests. In contrast, the advanced access appointment scheduling system accommodates all patients on the day of their request. The core idea is to schedule same-day appointments only [16]. Robinson and Chen [27] compare these two contrasting scheduling policies regarding various performance measures. Both appointment scheduling systems show a lack of planning security with respect to the actual number of treatments. The traditional appointment scheduling system is disadvantageous for two reasons: short-term cancellations and no-shows, i.e., patients who do not show up for their appointments. Overbooking strategies have been proven to reduce the resulting negative impacts [27–33]. Further difficulties for the traditional appointment scheduling system are, for example, long access times to appointments and insufficient time for (walk-in) patients with acute or urgent concerns [34]. The advanced access appointment scheduling system becomes less efficient due to the uncertain demand for treatment. This causes waiting times for patients as well as inefficient and imbalanced working hours for PCPs [27]. The request for appointments scheduled in advance is also not considered, which particularly affects elderly and chronically ill patients [25, 26]. Furthermore, on days with high demand for treatment patients are still referred to the next session or the following day—the so-called overflow patients. Thus, same-or-next-day scheduling policies also appear in the context of advanced access systems [16] which have been proven to be successful against simple same-day scheduling policies [27]. For this reason, hybrid appointment scheduling systems are introduced that combine the traditional and the advanced access appointment scheduling systems  [27, 31, 35]. Finally, the carve-out appointment scheduling system is managed as the traditional appointment scheduling system where a part of the PCP’s working hours is reserved for specific procedures or urgent services [25, 26]. Both the hybrid and carve-out appointment scheduling systems become less efficient if the ratio between traditional scheduled appointments, and same-day appointments or reserved working hours for urgent services is not chosen accurately. A disadvantage and a major challenge of these systems is therefore determining the correct ratio [27]. We note that the categorization of appointment scheduling systems becomes blurred as the systems overlap. In addition, the categorization is not clearly defined due to different assumptions, settings, services, and facilities of various health care systems of different countries.

Besides the differentiation in appointment scheduling systems, studies are further categorized according to intraday and interday appointment scheduling [36]. Intraday appointment scheduling concentrates on a single day with emphasis on, for example, the reduction of patient waiting times or the patient-physician continuity. In this context, mostly same-day scheduling policies are investigated on tactical and operational decision levels. In contrast, the concept of interday appointment scheduling aims at allocating appointments for consecutive days so that the PCP’s working hours are efficiently used. More precisely, on a tactical decision level, the question arises as to how many appointments are scheduled on which days in order to manage demand and take uncertainties such as walk-ins into account. Furthermore, fluctuating demand for treatment is smoothed throughout the week, and therefore, the utilization of the PCP’s working hours and patient arrivals are controlled in a more efficient way. The allocation and management of the PCP’s capacity is significant. This often includes the capacity ratio between same-day demand and demand that can be postponed.

In this study, we analyze an appointment scheduling system for a single PCP operating in a primary care practice that analogously operates to a carve-out system. Appointments are traditionally scheduled while ample time is reserved for walk-ins to serve their urgent needs. For designing an appointment scheduling system, we refer to studies [37, 38] that introduce a notion similar to our tactical scheduling planning. However, unlike these studies, we consider interday rather than intraday appointment scheduling and mainly focus on the balance of the PCP’s utilization. As we consider interday appointment scheduling in this study, we propose further related literature in the following section that focuses on the ratio of same-day demand to demand that can be postponed.

### Related work

Qu et al. [39] manage a hybrid appointment scheduling system by a two time horizon. The first time horizon is used to schedule same-day appointments and the second to postpone demand for treatment within a maximum of one week. Regarding the expectation and variance of the number of patients seen by the PCP, the authors show that the two time horizon system is at least as good as an advanced access appointment scheduling system. Further, Qu et al. [34] investigate the ratio between same-day appointments and appointments in advance by an analytical method with the aim of maximizing the expected number of treated patients. The results show that the optimal ratio depends on the PCP’s capacity, the ratio of the show-up rate of prescheduled appointments to the show-up rate of same-day appointments, and the joint distribution of demands for prescheduled and same-day appointments.

Dobson et al. [40] formulate a stochastic model to determine the optimal number of slots reserved for urgent patients. They compare the resulting carve-out appointment scheduling system with an advanced access appointment scheduling system on the basis of revenue, i.e., the average number of urgent patients who are not seen during the PCP’s working hours and the average queue length for routine patients. Feldman et al. [36] develop a dynamic appointment scheduling model that considers not only appointment cancellations and no-shows but also patients’ preferences. If appointments do not match their preferences, patients are not seen by the PCP. With their so-called next-*n* policy, Feldman et al. aim at a maximum expected revenue per day, which is determined by patients treated who are allowed to choose their appointments from *n* days.

**Fig. 1:**
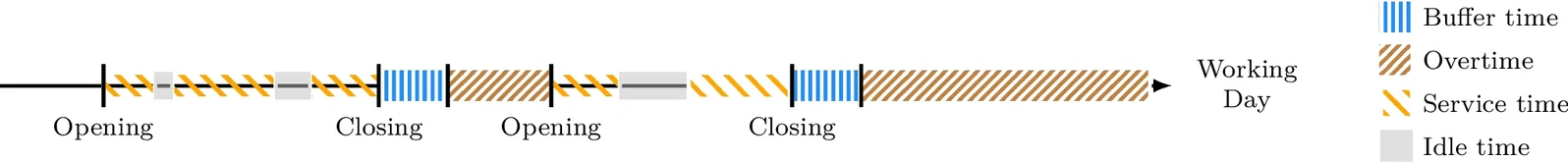
Schematic representation of a PCP’s morning and afternoon session visualizing buffer, idle- and overtime

In contrast to these studies, like Wiesche et al. [20], we consider patients with acute concerns who attempt scheduling an appointment within their willingness to wait. If no appointment is proposed within this time, they forgo scheduling an appointment and become walk-ins. This behavior is inspired by German practices where acute patients may walk into the practice even though there are no more appointments available. If possible, the patient is suggested a preferred time to visit the practice. However, we note that walk-ins have to expect long waiting times for their treatments as in the treatment order patients with appointments are preferred over walk-ins. In this study, we follow this strategy and postpone possible overbooking strategies to future studies. Wiesche et al. [20] determine the minimum number of appointments scheduled for a weekly profile to match capacity with demand by a mixed integer program formulation. Stochastic simulation shows that their appointment scheduling strategy balances the PCP’s work hours and reduces overflow patients by offering mainly walk-in blocks and only a few appointments before and after lunch. Schacht [41] extends the contributions by seasonal and stochastic aspects. He concludes that reconfiguration of the PCP’s working hours throughout the year is advantageous. Unlike studies of Wiesche et al. [20] and Schacht [41], we do not minimize the number of appointment slots to have as much capacity as possible for walk-ins. Instead, we endeavor to offer appointment slots, motivated by the German health care system where only three percent of all primary care practices do not offer appointments [19]. More precisely, we aim at determining an optimal daily ratio between appointment slots and reserved time for walk-ins to control the PCP’s workload to match the PCP’s capacity with demand for treatment. We also note that, unlike studies [20] and [41], we do not neglect patients whose demand for treatment is known well in advance, for instance as it is the case for chronically ill patients. Instead, we use this controllable demand to manage and balance the PCP’s workload.

The key difference between previously published studies and ours is that we use combinatorial optimization and do not consider stochastic but robust optimization models. More precisely, we use a robust network flow and design model. Network flow models are well analyzed and solvable in polynomial time [42, 43]. In contrast, robust flows with consistent flow constraints under demand uncertainty are $\mathcal{N}\mathcal{P}$-hard to compute [44]. To the best of our knowledge, we are the first who use combinatorial and robust solving methods to develop an appointment scheduling system.

## Setting and problem definition on tactical decision level

In this section, we introduce notations and formalize the appointment scheduling problem. In Section 3.1, we introduce a deterministic physician-patients setting. In Section 3.2, we show how to control demand for treatment by masked-based interday appointment scheduling. In Section 3.3, we present the MDP.

We note that the appointment scheduling problem considered in this section is on a tactical decision level and comparable with capacity allocation. We do not schedule individual patients to appointment slots but analyze the required capacity for walk-ins, chronic, and acute patients. For this reason, we consider the model on a higher level and do not take into account all events and uncertainties, such as scheduling times, service times, and cancellations. These mostly patient individual events and uncertainties are modeled on the operational level in the case study by agent-based simulation presented in Section 6.

### Deterministic physician-patients setting

Like most studies on primary health care [25], our study assumes a *single-server system*, i.e., practices with one PCP, because of the importance of patient-physician continuity. The single-server assumption holds even in joint practices, as PCPs usually provide medical care to their own panel of patients [24, 45]. Furthermore, we assume patient loyalty: patients seek medical care by their PCP and do not abandon them, for example by switching to another PCP. We note that multiple-server systems become important if the utilization of individual PCPs is not balanced and their capacities do not meet the demand for treatment. PCPs generally operate in clinical sessions—a morning and an afternoon session. We denote the set of all sessions by $\mathcal {K}$. Concentrating on a single working week, we describe each *session* by a tuple $k=(i,j)\in \mathcal {K}$, where $i\in \{0,\ldots ,4\}$ indicates the working day from Monday to Friday and a binary indicator $j\in \{0,1\}$ represents whether the morning (j=0) or afternoon (j=1) session is considered. For each of these sessions, we refine the PCP’s working hours by differentiating between opening hours, buffer time, and overtime. *Opening hours* determine the time span $\boldsymbol{o}\in \mathbb {Z}^{\mathcal {K}}_{\ge 0}$ in which appointments are scheduled and patients may be admitted for treatments. After opening hours, we assume PCPs to use *buffer time* $\boldsymbol{b}{}\in \mathbb {Z}^{\mathcal {K}}_{\ge 0}$ to compensate for contingencies such as delays. We refer to the time during the start of the opening hours and the end of the buffer as the PCP’s *capacity*, denoted by parameter $\boldsymbol{c} := \boldsymbol{o}\,+\,\boldsymbol{b}$. PCPs who do *overtime* work even beyond the buffer time. Figure 1 illustrates an exemplary working day of a PCP who operates in two sessions.

During the PCP’s opening hours, patients have two options to seek medical care in the modeled setting—either by appointment or as walk-in. Whether or not patients try to make an appointment or accept a proposed appointment depends on their health status and their *willingness to wait*. In this context, we divide patients into three categories: walk-ins, chronic patients, and acute patients.

*Walk-ins* have no willingness to wait, forgo scheduling an appointment, and walk into the practice during a specific session without prior notice. We specify their demand per session by parameter $\boldsymbol{d}^w \in \mathbb {Z}^{\mathcal {k}}_{\ge 0}$. *Chronic patients* request (recurring follow-up) appointments well in advance. We assume that their willingness to wait ensures flexibility in their appointment requests and we therefore specify their demand as a single weekly demand $d^{c}\in \mathbb {Z}_{\ge 0}$. *Acute patients* prefer an appointment and request one for a specific session, specified by demand $\boldsymbol{d}^a\in \mathbb {Z}^{\mathcal {K}}_{\ge 0}$. If they are not offered an appointment or an alternative appointment within their willingness to wait, acute patients walk into the practice during the session of their initial request and are handled like walk-ins. Motivated by the German labor law, which states that employees must provide a sick note after three days of sick leave [46], we uniformly set their willingness to wait to a maximum of three days. Thus, this is the period in which we want to guarantee an appointment for acute patients. We note that the performance of the appointment scheduling systems determines how many acute patients become walk-ins, in other words, how much controllable demand becomes uncontrollable demand. Nevertheless, there always exist acute patients who arrange appointments but do not attend them. As non-occurring demand only becomes known when the appointment would take place, we specify this missing demand per session by parameter $\boldsymbol{d}{}^n{}\in \mathbb {Z}^{\mathcal {K}}_{\ge 0}$ and refer to it as no-show demand.

If patients receive treatment, the duration of the treatments is subject to variation. Nevertheless, studies have shown that the length of the treatments only marginally varies in primary care [47, 48]. Thus, we assume a uniform *anticipated service time* $t\in \mathbb {Z}_{>0}$ for all patients.

Before proposing a method to manage the patient demand, we note that our models generalize to finer demand aggregations, for example, for every 30 minutes of a session. However, as our models support on a tactical decision level and as some of the models are based on a single demand realization, finer aggregations may lead to overfitting and consequently to appointment scheduling systems that are not generally applicable.

### Controlling patient demand by masked-based interday appointment scheduling

Naturally, patient demand is unevenly distributed over the sessions within a week. Addressing this issue, we manage controllable demand and consider potential uncontrollable demand by interday appointment scheduling. More precisely, we beneficially distribute chronic demand $d^{c}$ over the week while we shift acute demand $\boldsymbol{d}{}^a{}$ from days with high demand to days with lower demand. Following this strategy, we account for the walk-in demand $\boldsymbol{d}^{w}{}$ which is not controllable by postponement to a later session. To address this issue, we employ a cyclic model of a single working week. We assume that the acute demand which is shifted to the following week due to scheduled appointments, e.g., from Friday to Monday, compensate for the acute demand that would, theoretically, be shifted from the previous week. To shift (part of) the acute demand $d^a_k$, $k\in \mathcal {K}$ to an alternative session that is within the patient’s willingness to wait, we define a set of all feasible sessions as follows

$$\begin{aligned} \mathcal {K}^{+}_{k=(i,j} = \left\{ \begin{array}{ll} {k^{\prime }=(i^{\prime },j^{\prime })} \in \mathcal {K} \ \Big \vert \begin{array}{ll}& (i^{\prime }-i )\ \textrm{mod}\ 5\le 2, \\ &  o_{k^\prime }>0 \end{array} \end{array} \right\} . \end{aligned}$$

For later purposes, we define a set to summarize the sessions originally have been requested by acute patients alternatively scheduled in session ${k}\in \mathcal {K}$ as follows

$$\begin{aligned} \mathcal {K}^{-}_{{k}=(i,j)} = \left\{ \begin{array}{ll} \{k^{\prime }=(i^{\prime },j^{\prime }) \in \mathcal {K} \ \Big \vert \begin{array}{ll}& -(i^{\prime }-i)\ \textrm{mod}\ 5\le 2, \\ & O_{k^{\prime }}>0 \end{array} \end{array} \right\} . \end{aligned}$$

Using these sets, we formalize the distribution of the chronic demand and the shifts of the acute demand in the following definition.

#### Definition 1

**(Assignment)** Let demands $d^{c}\in \mathbb {Z}_{\ge 0}$ and $\boldsymbol{d}{}^a{}\in \mathbb {Z}^{\mathcal {K}}_{\ge 0}$ be given. A $(d,\boldsymbol{d}^{a})$-*assignment* $\boldsymbol{\alpha }=(\alpha ^{c}, \boldsymbol{\alpha }^{a}) = (\alpha ^{c}, \alpha _{(0,0)}^{a}, \ldots , \alpha _{(4,1)}^{a})$ is a $(|\mathcal {K}|+1)$-tuple of functions $\alpha ^{c}:\mathcal {K}\rightarrow \mathbb {Z}_{\ge 0}$ and $\alpha _{k}^{a}:\mathcal {K}^{+}_{k}\rightarrow \mathbb {Z}_{\ge 0}$, $k\in \mathcal {K}$ that distribute and shift the demand across sessions, respectively, such that $\sum _{k\in \mathcal {K}}\alpha ^{c}(k)=d^{c}$ and $\sum _{k^\prime \in \mathcal {K}^{+}_{k}}{\alpha _{k}^{a}}(k^\prime ) \le {d_{k}^{a}}$, $k\in \mathcal {K}$ holds.

Considering the definition of an assignment, we note the following three aspects. First, an assignment has to distribute the entire chronic demand $d^c$ and may not shift more than the acute demand $d^{a}_k$ of each session $k\in \mathcal {K}$. Second, whenever an acute patient is assigned by function $\boldsymbol{\alpha }^{a}$ to the requested or a later occurring session, we assume that the patient will see the PCP by appointment. Conversely, this means that the number of acute patients who are not assigned, i.e., ${d_{k}^{a}}-\sum _{k^\prime \in \mathcal {K}^{+}_{k}}{\alpha _{k}^{a}}(k^\prime )$, determines the number of acute patients who become walk-ins in session $k\in \mathcal {K}$. Third, assignments do not have to respect the PCP’s capacity by definition and thus may lead to overtime. For this reason, we strive for a system by which demand is reasonably assigned—in other words, an appointment scheduling system.

**Fig. 2:**
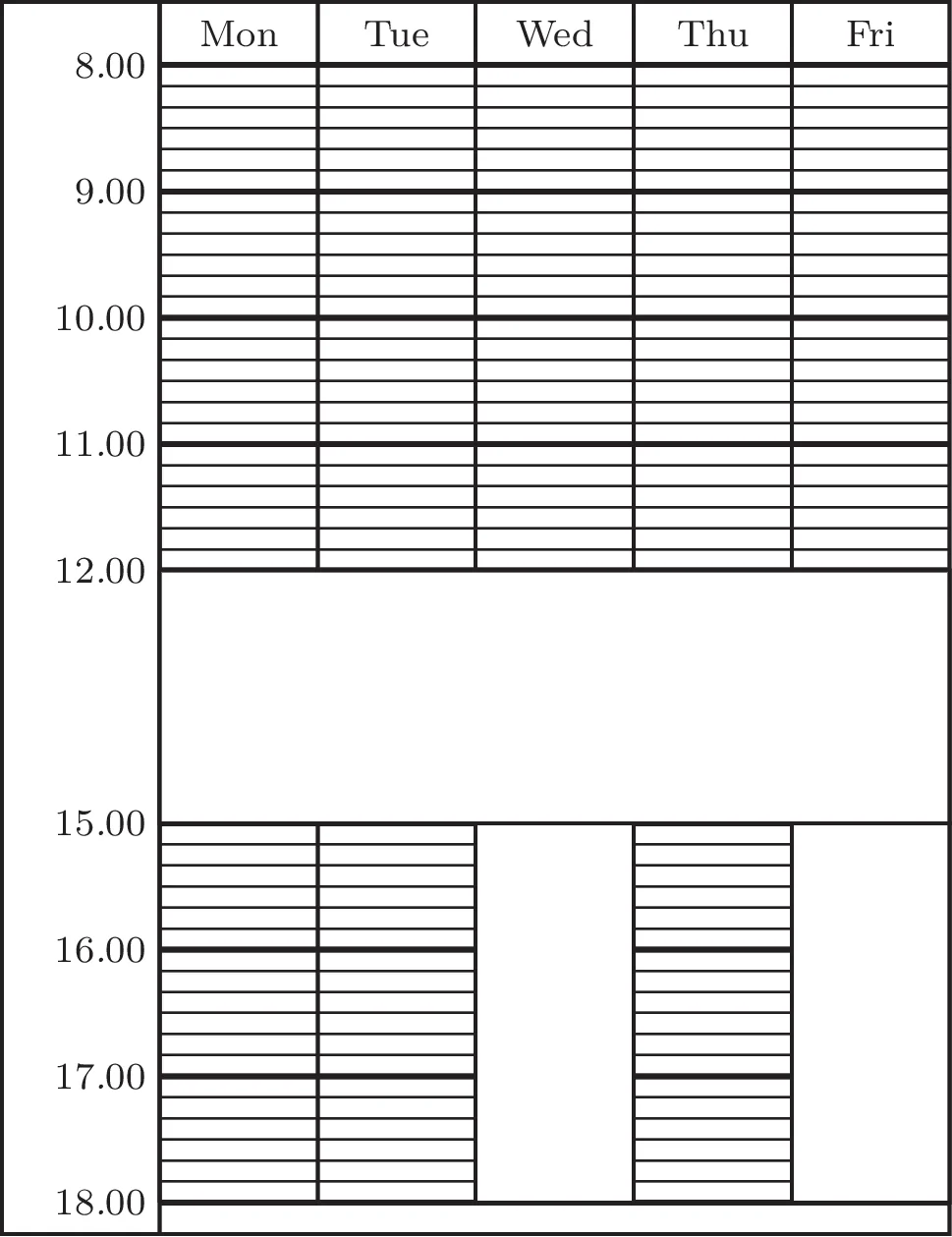
Schedule defined by consecutive slots

In primary care practices, appointment scheduling systems are usually based on time slots. To implement time slots in our setting, we divide the time span $o_{k}$ of the PCP’s opening hours for each session $k\in \mathcal {K}$ into time periods of equal length $\ell \in \mathbb {Z}_{>0}$. Each *slot* is described by a tuple $s=(k,q_{k})$ where $k\in \mathcal {K}$ indicates the session and $q_k\in \{1,\ldots , n_{k} \}$ with $n_{k}:=\lceil \frac{o_{k}}{\ell }\rceil \in \mathbb {Z}_{\ge 0}$ the position. We denote the set of all slots within the weekly PCP’s capacity by $\mathcal {S}$. We assume one scheduled patient per slot and, in order to reserve a reasonable amount of time, we require the slot length ℓ to be at least as long as the anticipated service time t, i.e., $\ell \ge t$. Figure 2 visualizes an exemplary schedule of a five-day working week with ten-minute slots where the PCP’s practice is closed on Wednesday and Friday afternoons.

At this point, we allow slots to have one of the following three *states*: chronic c, acute a and walk-in w. States are supposed to indicate whether a slot is reserved to schedule a chronic or acute patient or whether a slot is reserved for a potential walk-in. For simplicity’s sake, we refer to slots with state ‘chronic’, ‘acute’, and ‘walk-in’ as chronic, acute, and walk-in slots. Using the states of slots, we can define a mask.

#### Definition 2

**(Mask)** A *mask* is a function $\mu :\mathcal {S}\rightarrow \{c, a, w\}$ that assigns each slot one state.

The aim is to assign demand, or in other words, schedule appointments on the basis of a mask. As a mask regulates how many chronic and acute patients may be scheduled and how many slots are reserved for potential walk-ins, an assignment needs to be compatible with the considered mask.

#### Definition 3

**(**$\boldsymbol{\alpha }$**-compatible mask)** Mask μ and $(d^{c},\boldsymbol{d}^{a})$-assignment $\boldsymbol{\alpha }=(\alpha ^{c}, \boldsymbol{\alpha }^{a})$ are *compatible* with each other if $\boldsymbol{\alpha }$ assigns at most as much chronic and acute demand as chronic and acute slots are available, respectively, i.e., for $k\in \mathcal {K}$ it holds

$$\begin{aligned} \alpha ^{c}(k)&\le n_{\mu }(c,k) := \left| \{s=(k,q_k)\in \mathcal {S}\mid \mu (s)=c\}\right| ,\\ \sum _{k^\prime \in \mathcal {K}^{-}_{k}}{\alpha }_{k^\prime }^{a}(k)&\le n_{\mu }(a,k) := \left| \{s=(k,q_k)\in \mathcal {S}\mid \mu (s)=a\}\right| . \end{aligned}$$

The set of all $(d^{c},\boldsymbol{d}^{a})$-assignments that are compatible with mask μ is denoted by $\mathcal {A}^{\mu }$. If $\boldsymbol{\alpha }\in \mathcal {A}^{\mu }$ holds, we say a mask μ is $\boldsymbol{\alpha }$*-compatible*.

Overall, we obtain an appointment scheduling system based on a mask and compatible assignments referred to as *mask-based appointment scheduling system*. In the next section, we design a mask that aims at a balanced utilization of the capacity of the PCP. We note that masks can be designed according to different objectives such as the distribution of certain types of patients to certain working days.

### Balanced utilization by the MDP

One of the main goal PCPs strive for is an efficient workflow. This includes a balanced workload to avoid PCPs working overtime on one day while not working at full capacity on another day of the same week. To achieve this in our setting on a tactical decision level, we strive for a balanced use of the weekly capacity. We start with formal definitions for the workload per session, the weekly utilization, and the optimal workload per session in terms of a balanced utilization of the weekly capacity.

**Table 1:** Notation table

Notation	Specification
$\mathcal {K}$	index set of sessions
$\mathcal {S}$	set of slots
$\mathcal {A}^{\mu }$	set of assignments suitable for mask μ
$d^{c}$	weekly chronic demand
${d_{k}^{a}}$	acute demand per session $k\in \mathcal {K}$
$d_{k}^{a}$	walk-in demand per session $k\in \mathcal {K}$
$d_{k}^{n}$	no-show demand per session $k\in \mathcal {K}$
$o_{k}$	PCP’s opening hours in session $k\in \mathcal {K}$
$$n_{k} $$	number of slots in session $$k\in \mathcal {K}$$
$b_{k}$	PCP’s buffer time in session $k\in \mathcal {K}$
$c_{k}$	PCP’s capacity in session $k\in \mathcal {K}$
C	PCP’s weekly capacity
t	anticipated service time
ℓ	slot length
$u^{}_{}$	PCP’s utilization
$\alpha ^{c}$	weekly assignment for chronic patients
${\alpha _{k}^{a}}$	assignment for acute pat. from session $k\in \mathcal {K}$
$\boldsymbol{\alpha }$	demand assignment
$n_{\mu }(c,k)$	number of chronic slots in mask μ
$n_{\mu }(a,k)$	number of acute slots in mask μ
$\varPhi _{k}^{\boldsymbol{\alpha }}$	PCP’s workload under assignment $\boldsymbol{\alpha }$ and
	demands of session $k\in \mathcal {K}$

**Fig. 3:**
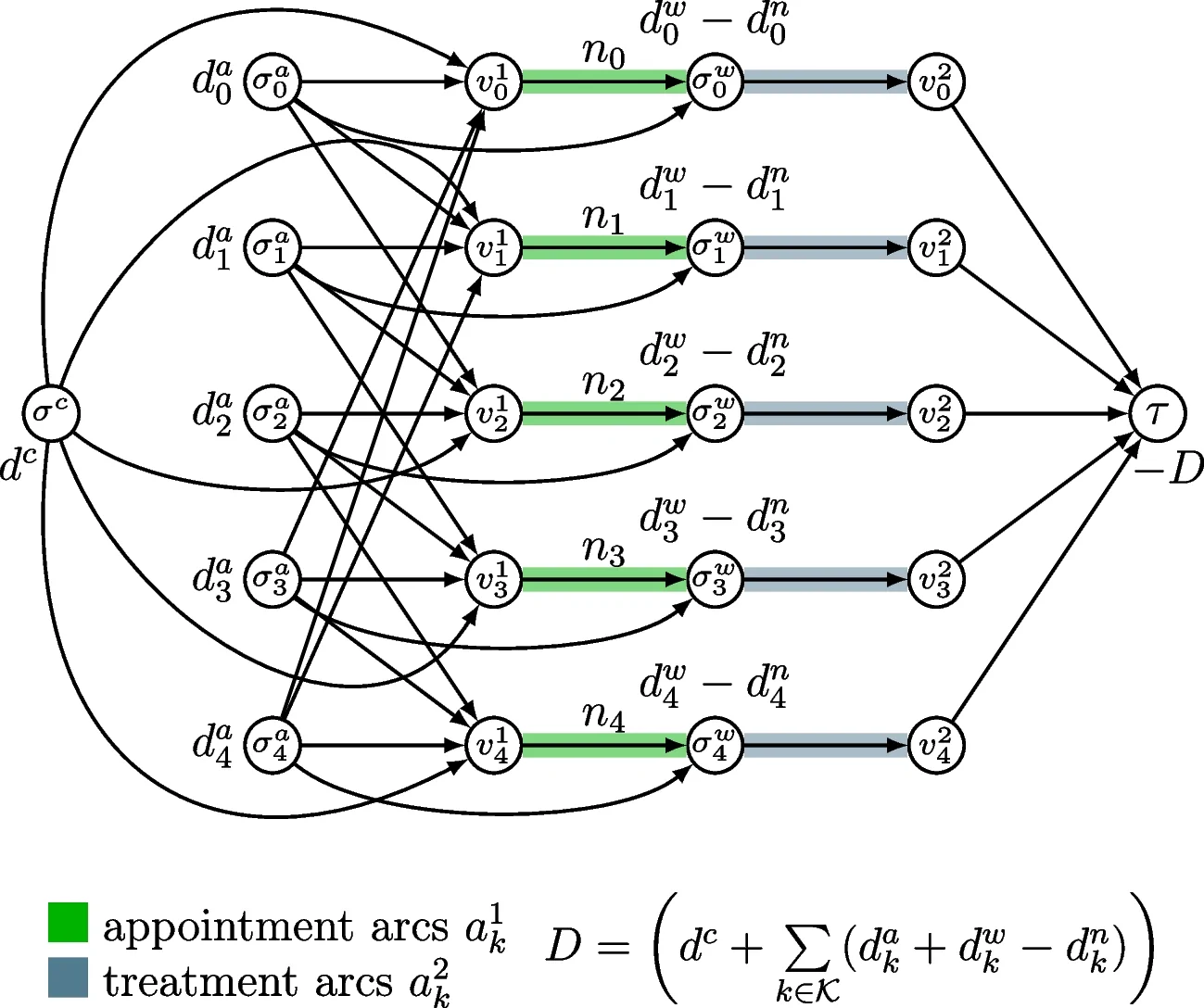
Network flow model for managing patient flow

Given demands $d^{c}\in \mathbb {Z}_{\ge 0}$ and $\boldsymbol{d}^{a}, \boldsymbol{d}^{w}, {\boldsymbol{d}^{n}}\in \mathbb {Z}_{\ge 0}^{\mathcal {K}}$ as well as a $(d^{c}, \boldsymbol{d}^{a})$-assignment $\boldsymbol{\alpha }$, we define the PCP’s *workload* $\varPhi _{k}^{\boldsymbol{\alpha }}$ in session $k\in \mathcal {K}$ by the anticipated working time resulting from all treatments requested in and assigned to session k minus no-shows, i.e.,

$$\begin{aligned} \varPhi _{k}^{\boldsymbol{\alpha }} =&\left( d_{k}^{a} + {d_{k}^{a}} - \sum \limits _{k^\prime \in \mathcal {K}^{+}_{k}}{\alpha _{k}^{a}}(k^\prime ) {-d_{k}^{n}}\right. \\&\left. \hspace{1.525cm}+\sum \limits _{k^\prime \in \mathcal {K}^{-}_{k}}{\alpha }_{k^\prime }^{a}(k) +\alpha ^{c}(k) \right) t. \end{aligned}$$

We note that the PCP’s workload depends on demands and assignments. Recalling the cyclic model and using the weekly patient demand, we can compute the *weekly utilization *$u^{}_{}$ of the PCP by

$$ u   = \frac{t}{C}\left( d^{c}+\sum \limits _{k\in \mathcal {K}}({d_{k}^{a}} + d_{k}^{a}{-d_{k}^{n}})\right) , $$

where $C=\sum \limits _{k\in \mathcal {K}}c_{k}$ denotes the PCP’s weekly capacity. We obtain the, in terms of balance, *optimal workload* per session as the product of the weekly utilization and the session’s capacity, i.e., $u\cdot c_k$ for $k\in \mathcal {K}$. As the PCP’s workload depends on assignments and the assignments in turn are restricted by masks, we can manage the PCP’s workload by the design of a mask. Aiming at an optimal workload in every session, we evaluate masks according to the achievable workload balance.

#### Definition 4

Let the PCP’s capacity $\boldsymbol{c}\in \mathbb {Z}^{\mathcal {K}}_{\ge 0}$ and demands $d^{c}\in \mathbb {Z}_{\ge 0}$, $\boldsymbol{d}^{a}, \boldsymbol{d}^{w}{,\boldsymbol{d}^{n}}\in \mathbb {Z}_{\ge 0}^{\mathcal {K}}$ be given. We define the *cost* of a mask μ as the maximum deviation of the actual from the optimal workload among all sessions, provided that the workload is subject to a $(d^{c}, \boldsymbol{d}^{a})$-assignment $\boldsymbol{\alpha }\in \mathcal {A}^{\mu }$ that minimizes the cost, i.e.,

$$ c(\mu ) = \min _{\boldsymbol{\alpha }\in \mathcal {A}^{\mu }} \max _{k\in \mathcal {K}} \left| \varPhi _{k}^{\boldsymbol{\alpha }} - u   \cdot c_{k} \right| . $$

We conclude this section with the definition of the MDP and a summary of notations in Table 1.

#### Definition 5

**(MDP)** Given the PCP’s capacity $\boldsymbol{c}\in \mathbb {Z}^{\mathcal {K}}_{\ge 0}$ and demands $d^{c}\in \mathbb {Z}_{\ge 0}$, $\boldsymbol{d}^{a}, \boldsymbol{d}^{w}{,\boldsymbol{d}^{n}}\in \mathbb {Z}_{\ge 0}^{\mathcal {K}}$, the *mask design problem* aims at a mask of minimum cost.

## Modeling the MDP

In this section, we present an extensive combinatorial interpretation of the MDP by a network flow and design model. First, we consider a model in a deterministic setting and subsequently extend this model to a robust setting.

### MDP in a deterministic setting

In this section, we model the MDP by a network flow and design formulation. For this purpose, we construct a network such that a flow represents a PCP’s patient flow of one week from request to treatment with or without appointment. An exemplary network of a five-day working week with one session per day is visualized in Fig. 3. The network is defined by an acyclic digraph G=(V,A) with vertex set *V* and arc set *A*. Besides vertices $v^1_k$ and $v^2_k$ for all $k\in \mathcal {K}$, vertex set *V* includes multiple sources and sinks that represent the patient demand. More precisely, let $\sigma ^{c}_{} \in V$ be the source that models the weekly chronic demand and let $\sigma ^{a}_{k} \in V$ be the source that models the acute demand per session $k\in \mathcal {K}$. Vertex $\sigma ^{w}_{k} \in V$ models the demand per session $k\in \mathcal {K}$ which results from walk-ins minus no-shows. Let $\tau \in V$ be the sink which ensures that all patients are seen by the PCP. A flow originating from one of the sources only reaches sink $\tau \in V$ by using at least one of two kind of arcs. The first type of arcs $a^1_k= (v^1_k, \sigma ^{w}_{k} )\in A$ represents acute and chronic patients scheduled in session $k\in \mathcal {K}$ referred to as appointment arcs. To limit the number of patients scheduled, we define upper arc capacities $\overline{\psi }_{} :A\rightarrow \mathbb {Z}_{\ge 0}$ on the appointment arcs that are equal to the number of slots of the PCP’s opening hours, i.e., $\overline{\psi }_{} (a^1_k)=n_{k}$ for all $k\in \mathcal {K}$. As acute patients may be scheduled within three days after their requests, we add arcs that connect source $\sigma ^{a}_{k}$, $k\in \mathcal {K}$ with the tail of the appointment arcs, i.e., with vertices $v^1_{k^\prime }\in V$ for $k^\prime \in \mathcal {K}^{+}_{k}$. Furthermore, as chronic patients may be scheduled in each session, source $\sigma ^{c}_{}$ is also connected with vertex $v^1_k$ for all $k\in \mathcal {K}$. The second type of arcs $a^2_k= ( \sigma ^{w}_{k} , v^2_k) \in A$ represents all patients seen by the PCP in session $k\in \mathcal {K}$ referred to as treatment arcs. Accordingly, the treatment arc $a^2_k$ is connected at vertex $\sigma ^{w}_{k} \in V$, $k\in \mathcal {K}$ in series with the appointment arc $a^1_k$ of the same session $k\in \mathcal {K}$. In addition, source $\sigma ^{a}_{k}$, $k\in \mathcal {K}$ is also connected with vertex $\sigma ^{w}_{k}$, i.e., with the tail of treatment arc $a^2_k$, which models acute patients who become walk-ins. Finally, each treatment arc $a^2_k$, $k\in \mathcal {K}$ is connected with sink τ. For all vertices v∈V, balances $\beta :V\rightarrow \mathbb {Z}$ with $\sum _{v\in V} \beta (v) =0$ are defined as follows

$$\begin{aligned} \beta (v)= {\left\{ \begin{array}{ll} d_{k}^{a} &  \text { if } v= \sigma ^{a}_{k} , k\in \mathcal {K}, \\ d_{k}^{w}{-d_{k}^{n} }& \text { if } v= \sigma ^{w}_{k} , k\in \mathcal {K}, \\ d^{c}& \text { if } v= \sigma ^{c}_{} ,\\ -\left( d^{c}+\sum \limits _{k\in \mathcal {K}}\left( d_{k}^{a}+d_{k}^{w}\right) \right) & \text { if } v= \tau , \\ 0 & \text { otherwise.} \end{array}\right. } \end{aligned}$$

Overall, we obtain network $N =( G , \overline{\psi }_{,} \beta )$.

In network *N*, a feasible β*-flow* is defined by a function $f:A\rightarrow \mathbb {Z}_{\ge 0}$ that satisfies the flow balance constraints $\sum _{a=(v,w)\in A} f(a) - \sum _{a=(w,v)\in A} f(a) = \beta (v)$ at every vertex v∈V and the capacity constraints $0 \le f(a^1_k) \le \overline{\psi }_{} (a^1_k)$ on all appointment arcs $a^1_k\in A$. We note that all flows in this study are defined to be integral. A β-flow in network *N* corresponds to a $(d^{c}, \boldsymbol{d}^{a})$-assignment and a mask that are compatible with each other. We recall that the flow value on the treatment arc $a^2_k$ represents the number of patients seen by the PCP in session $k\in \mathcal {K}$. So, multiplying this number by the anticipated service time t results in the PCP’s workload in session $k\in \mathcal {K}$. Thus, to obtain an optimal solution to the MDP, we aim at a feasible β-flow *f* in network *N* that is balanced on the treatment arcs, i.e., a flow *f* that minimizes the following expression

1 $$\begin{aligned} \max _{k\in \mathcal {K}} | t\cdot f(a^2_k) - u^{}_{} \cdot c_{k}|. \end{aligned}$$

To determine such a flow, we add lower and upper arc capacities $\underline{\psi }_{\omega } , \overline{\psi }_{\omega } :A\rightarrow \mathbb {Z}_{\ge 0}$ for a parameter $\omega \in \mathbb {Z}_{\ge 0}$ on the treatment arcs $a^2_k\in A$, $k\in \mathcal {K}$ of network *N*. The lower and upper arc capacities are defined such that the PCP’s actual workload in session $k\in \mathcal {K}$ may deviate from the optimal workload $u^{}_{} \cdot c_{k}$ by an amount of $\omega \in \mathbb {Z}_{\ge 0}$, i.e., $\underline{\psi }_{\omega } (a^{2}_k)=\max \Big \{\Big \lceil \frac{1}{t} \left( u^{}_{} \cdot c_{k}-\omega \right) \Big \rceil , 0\Big \}$, $\overline{\psi }_{\omega } (a^{2}_k)=\Big \lfloor \frac{1}{t}\left( u^{}_{} \cdot c_{k}+\omega \right) \Big \rfloor .$ We denote the resulting network by $N _\omega =( G , \overline{\psi }_{} , \overline{\psi }_{\omega } , \underline{\psi }_{\omega } , \beta )$. If parameter ω is minimal, a feasible β-flow in network $N _\omega$ corresponds to a feasible flow that minimizes expression (1). Consequently, for minimum parameter ω, a feasible β-flow in network $N _\omega$ defines an optimal solution to the MDP.

Before presenting an algorithm to find the minimum parameter ω, we consider the solution values that can be realized in the MDP. The PCP’s workload $\varPhi _{k}^{\boldsymbol{\alpha }}$ is computed by the anticipated service time t times the number of treatments in session $k\in \mathcal {K}$ which can vary between zero and $D_k:= d_{k}^{a} {-d_{k}^{n}}+ \sum _{k^\prime \in \mathcal {K}^{-}_{k}}d_{k^\prime }^{a} + d^{c}$. Thus, the possible solution values to the MDP form the finite set $\varOmega = \bigcup _{k\in \mathcal {K}} \varOmega _k$ with

$$ \varOmega _k= \left\{ | t\cdot i - u   \cdot c_{k}| \big \vert i\in \left\{ 0,1,\ldots ,D_k\right\} \right\} . $$

Using this, Algorithm 1 attached to Appendix A computes the optimal parameter $\omega \in \varOmega$ based on the following procedure. For the sake of simplicity, binary search is used to first determine the minimum parameter *i* of set $\{0,1,[3]\ldots ,\max _{k\in \mathcal {K}} D_k\}$ for which a feasible β-flow exists in network $N _{\overline{\omega }}$ with $\overline{\omega }= i\cdot t$. We note that the gap between two consecutive values in set $\varOmega _k$, $k\in \mathcal {K}$ is *t* (if the elements of set $\varOmega _k$ were ordered by size). Consequently, from the first step, we obtain an interval $(\overline{\omega }-t, \overline{\omega }]$ that contains the optimal parameter ω. In the second step, set Ω is searched for parameters that are within this interval (and smaller than $\overline{\omega }$), i.e., set $(\overline{\omega }-t, \overline{\omega })\cap \varOmega$ is searched. We note that such parameters may exist as the optimal workload $u^{}_{} \cdot c_{k}$ need not be a multiple of the anticipated service time t. If there are such parameters for which additionally feasible flows in the corresponding networks exist, the minimum parameter found so far is updated. We note that a feasible β-flow can be computed by, for example, the Minimum Mean Cycle Canceling algorithm in polynomial time [43]. Finally, Algorithm 1 returns a β-flow with minimum parameter $\omega \in \varOmega$ that defines an optimal solution to the MDP as shown in the following theorem.

#### Theorem 1

A mask determined by a β-flow obtained by Algorithm 1 is of minimum cost.

The proof is moved to Appendix A. We note that the flow obtained by Algorithm 1 is not unique, as for minimum parameter $\omega \in \varOmega$ an arbitrary feasible β-flow in network $N_\omega$ is determined. Using this, we integrate a second optimization stage into our network flow model that takes into account the following. Acute patients strive for an appointment. If they are offered an appointment within their willingness to wait, not only their requests are satisfied but also demand is beneficially controlled for the PCP. Considering this win-win situation, we aim at minimizing the unsuccessful appointment requests, or in other words, the number of acute patients who become walk-ins. Acute patients who become walk-ins have not been assigned by a $(d^{c},\boldsymbol{d}^{a})$-assignment $\boldsymbol{\alpha }=(\alpha ^{c}, \boldsymbol{\alpha }^{a})$. In total, that is $d_{w}^{a}(\boldsymbol{\alpha }^{a}):=\sum _{k\in \mathcal {K}}{d_{k}^{a}}-\sum _{k^\prime \in \mathcal {K}^{+}_{k}}{\alpha _{k}^{a}}(k^\prime )$ acute patients. As assignments are restricted by masks, the number of acute patients who become walk-ins is also influenced by the design of a mask. For our network flow model, this means that we aim at finding a β-flow in network $N _{\omega }$ with minimum flow value on arcs $( \sigma ^{a}_{k} , \sigma ^{w}_{k} )\in A$, $k\in \mathcal {K}$. We can easily implement this into our model by assigning arbitrary positive cost to these arcs. Overall, we obtain a minimum cost flow model that we integrate into Algorithm 1 by determining a minimum cost β-flow instead of an arbitrary feasible β-flow in the respective network. We refer to the model and procedure presented in this section as *Det-F*. We conclude this section with the following corollary.

#### Corollary 1

Det-F is a modeling for the MDP.

### MDP in a robust setting

So far, we considered the MDP in a deterministic setting based on the assumption that the entire demand data is given. However, as acute patients contact their PCP when they suffer from acute illnesses, their demand is subject to uncertainty. Naturally, the walk-in and no-show demand is also subject to uncertainty. Only the chronic demand is regulated by recurring appointments scheduled in advance. For an effective appointment scheduling system, a mask that is robust against demand uncertainties is essential, i.e., a mask whose use achieves a balanced utilization of the PCP’s capacity for all potential demands. Following the core idea of robust optimization, we aim at a version of the MDP whose solution is immune to variation in demand data. To address this issue, we model the uncertainty in acute, walk-in, and no-show demands by a set of scenarios. Our choice is a finite scenario set Λ due to the lack of historical data and the difficulty of, for example, budget uncertainty sets in terms of construction and computational complexity. In scenario $\lambda \in \varLambda$, the acute, walk-in, and no-show demands are specified by parameters $\boldsymbol{d}_{\lambda }^{a}\in \mathbb {Z}^{\mathcal {K}}_{\ge 0}$, $\boldsymbol{d}_{\lambda }^{w}\in \mathbb {Z}^{\mathcal {K}}_{\ge 0}$, and $\boldsymbol{d}_{\lambda }^{n}\in \mathbb {Z}^{\mathcal {K}}_{\ge 0}$, respectively. However, we assume that the variations in demand are limited due to reasons such as a fixed panel of patients. We require the total weekly acute, walk-in, and no-show demands to be equal for all scenarios, i.e., $\sum _{k\in \mathcal {K}} d_{\lambda ,k}^{a} = D^a$, $\sum _{k\in \mathcal {K}} d_{\lambda ,k}^{w} = D^w$, and $\sum _{k\in \mathcal {K}} d_{\lambda ,k}^{n} = D^n$ for all $\lambda \in \varLambda$ for given parameters $D^a, D^w, D^n\in \mathbb {Z}_{\ge 0}$. We note that the chronic demand is still specified by parameter $d^{c}$. Given this new setting, we define a mask that is robust to uncertainties in demand.

#### Definition 6

**(Robust Mask)** A mask is called *robust* if there exists a compatible $(d^{c},\boldsymbol{d}_{\lambda }^{a})$-assignment for each scenario $\lambda \in \varLambda$. The set of all $(d^{c},\boldsymbol{d}_{\lambda }^{a})$-assignments that are compatible with mask μ is denoted by $\mathcal {A}^{\mu }_\lambda$ for every scenario $\lambda \in \varLambda$.

We note that the robustness of a mask is by definition independent of the walk-in and no-show demand $\boldsymbol{d}_{\lambda }^{w}$ and $\boldsymbol{d}_{\lambda }^{n}$, $\lambda \in \varLambda$. Furthermore, we note that the PCP’s utilization $u^{}_{}$ and the resulting optimal workload $u^{}_{} \cdot c_{k}$ in session $k\in \mathcal {K}$ is independent of the scenario considered by assumption. In contrast, the PCP’s workload $\varPhi _{\lambda ,k}^{\boldsymbol{\alpha }}$ in session $k\in \mathcal {K}$ depends on a $(d^{c}, \boldsymbol{d}_{\lambda }^{a})$-assignment $\boldsymbol{\alpha }$ and on walk-in and no-show demands $d_{\lambda ,k}^{w}$ and $d_{\lambda ,k}^{n}$ and thus on scenario $\lambda \in \varLambda$.

Considering the set of scenarios, we evaluate masks according to the achievable workload balance in the worst-case scenario as formalized in the following definition.

#### Definition 7

Let the PCP’s capacity $\boldsymbol{c}\in \mathbb {Z}^{\mathcal {K}}_{\ge 0}$, demand $d^{c}\in \mathbb {Z}_{\ge 0}$, and demands $\boldsymbol{d}_{\lambda }^{a}, \boldsymbol{d}_{\lambda }^{w}, {\boldsymbol{d}_{\lambda }^{n}}\in \mathbb {Z}^{\mathcal {K}}_{\ge 0}$ for all scenarios $\lambda \in \varLambda$ be given. The *cost* of a robust mask μ is defined by the worst-case maximum deviation of the actual from the optimal workload among all sessions of all scenarios, i.e.,

$$ c(\mu )= \max _{\lambda \in \varLambda } \min _{\boldsymbol{\alpha }\in \mathcal {A}^{\mu }_\lambda } \max _{k\in \mathcal {K}} \left| \varPhi _{\lambda ,k}^{\boldsymbol{\alpha }} - u   \cdot c_{k} \right| . $$

Using this, we define a robust version of the MDP as follows.

#### Definition 8

**(rMDP)** Let $\boldsymbol{c}\in \mathbb {Z}^{\mathcal {K}}_{\ge 0}$ be the PCP’s capacity, $d^{c}\in \mathbb {Z}_{\ge 0}$ be the chronic demand, and $\boldsymbol{d}_{\lambda }^{a}, \boldsymbol{d}_{\lambda }^{w}, {\boldsymbol{d}_{\lambda }^{n}}\in \mathbb {Z}^{\mathcal {K}}_{\ge 0}$ be the acute, walk-in, and no-show demand for all scenarios $\lambda \in \varLambda$, respectively. The *robust mask design problem* aims at a robust mask of minimum cost.

In the next step, we model the rMDP by an adjusted network flow and design model in which a so-called robust flow under consistent flow constraints is sought [44]. We consider a network $N ^\varLambda =( G =(V,A), \overline{\psi }_{,} \boldsymbol{ \beta })$ whose balances $\beta ^\lambda :V\rightarrow \mathbb {Z}$ with $\sum _{v\in V} \beta ^\lambda (v)=0$ are given for all scenarios $\lambda \in \varLambda$, denoted by $\boldsymbol{ \beta }=( \beta ^{1},\ldots , \beta ^{{|\varLambda |}})$. Furthermore, we introduce two arc subsets $A_{\text {fix}}^c:=\{( \sigma ^{c}_{} , v^1_k) \mid k\in \mathcal {K}\}\subseteq A$ and $A_{\text {fix}}^a:= \{a^1_k\mid k\in \mathcal {K} \}\subseteq A$ and refer to their arcs as *fixed arcs*. In network $N ^\varLambda$, a feasible *robust* $\boldsymbol{ \beta }$*-flow* $\boldsymbol{f}=(f^1,\ldots , f^{|\varLambda |})$ is defined by feasible $\beta ^{\lambda }$-flows $f^\lambda : A\rightarrow \mathbb {Z}_{\ge 0}$ for all scenarios $\lambda \in \varLambda$ that satisfy the following equal flow property

$$\begin{aligned} (F) \hspace{0.25cm}&f^\lambda (a)=f^{\lambda ^\prime }(a) \text { for all } a\in A_{\text {fix}}^c\cup A_{\text {fix}}^a, \ \lambda , \lambda ^\prime \in \varLambda . \end{aligned}$$

Analogous to Section 4.1, a robust $\boldsymbol{ \beta }$-flow in network $N^{\varLambda }$ corresponds to a solution to the rMDP. To obtain an optimal solution, we aim at a feasible robust $\boldsymbol{ \beta }$-flow $\boldsymbol{f}=(f^1,\ldots ,f^{|\Lambda |})$ in network $N^{\varLambda }$ that minimizes the following expression

2 $$\begin{aligned} \max _{\lambda \in \varLambda } \max _{k\in \mathcal {K}} | t\cdot f^{\lambda }(a^2_k) - u^{}_{} \cdot c_{k} |. \end{aligned}$$

By an adjusted version of Algorithm 1, we can determine the desired robust $\boldsymbol{ \beta }$-flow $\boldsymbol{f}$ in network $N ^{\varLambda }_\omega =( G , \overline{\psi }_{} , \overline{\psi }_{\omega } , \underline{\psi }_{\omega } , \boldsymbol{ \beta })$ with minimum parameter $\omega \in \varOmega$, which additionally minimizes the unsuccessful treatment requests. However, we note that there generally does not exist a polynomial-time algorithm to compute a feasible robust flow on acyclic digraphs [44]. For this reason, we determine the robust flows computed in the adjusted algorithm by means of the following compact IP formulation.

3 $$\begin{aligned} \min&\ \ {\sum _{\lambda \in \varLambda }\sum _{k\in \mathcal {K}}\sum _{( \sigma ^{a}_{k} , \sigma ^{w}_{k} )\in A}f_{a}^{\lambda } } \end{aligned}$$

4 $$\begin{aligned}   [1em] \text {s.t. }&\sum _{a=(v,w)\in A} f_{a}^{\lambda } - \sum _{a=(w,v)\in A} f_{a}^{\lambda } =\beta ^{\lambda }(v)  &   \forall \ v \in V, \ \lambda \in \varLambda \end{aligned}$$

5 $$\begin{aligned}   [1em]&0\le f_{a}^{\lambda } \le \psi (a)  &   \forall \ a \in A, \lambda \in \varLambda \end{aligned}$$

6 $$\begin{aligned}   [1em]&{ \underline{\psi }_{\omega } (a)\le f_{a}^{\lambda } \le \overline{\psi }_{\omega } (a) }  &   \forall \ a=a_2^k \in A, \lambda \in \varLambda \end{aligned}$$

7 $$\begin{aligned}   [1em]&f_{a}^{\lambda } = f_{a}^{\lambda ^{\prime }}  &   \forall \ a \in A_{\text {fix}}^a\cup A_{\text {fix}}^c, \ \lambda , \lambda ^\prime \in \varLambda \end{aligned}$$

8 $$\begin{aligned}   [1em]&f_{a}^{\lambda } \in \mathbb {Z}_{\ge 0 }  &   \forall \ a \in A, \lambda \in \varLambda \end{aligned}$$

Let $f_{a}^{\lambda }\in \mathbb {Z}_{\ge 0}$ be the variables that model the flow on arc a∈A in scenario $\lambda \in \varLambda$. The IP can be formulated as presented in Equations 3–8. Objective (3) minimizes the flow value that represents unsuccessful treatment requests. The flow balance constraints (4) and capacity constraints (5) ensure feasible flows in all scenarios. Capacity constraints (6) guarantee the desired balanced flow on the treatment arcs. The so-called consistent flow constraints (7) enforce the desired equal flow values on fixed arcs. Constraints (8) define the domain of the variables.

Overall, we obtain the following theorem.

#### Theorem 2

A robust mask determined by a robust $\boldsymbol{ \beta }$-flow obtained by the adjusted Algorithm 1 is of minimum cost.

#### Proof

Analogous to the proof of Theorem 1.□

We refer to the resulting model and procedure presented in this section as *Rob-F* and conclude with the following corollary.

#### Corollary 2

Rob-F is a modeling for the rMDP.

## Robust multimask design problem and its modeling

In the previous section, we have considered the rMDP where the weekly distribution of the acute, walk-in, and no-show demands are uncertain, but the respective total demand remains the same in every scenario. Naturally, not only the distribution but also the total demand is uncertain and fluctuates. Under such circumstances, a robust mask may produce poor results with respect to our objective. For example, if many slots need to be reserved for appointments for acute patients in one scenario due to high demand and only a few in another scenario due to low demand, a robust mask would achieve a trade-off to minimize the worst-case scenario. This could lead to poor results for both scenarios, in contrast to a mask that is adapted to the level of demand. To address this issue, from now on we assume that the acute, walk-in, and no-show demands are specified by parameters $\boldsymbol{d}_{\lambda }^{a}\in \mathbb {Z}^{\mathcal {K}}_{\ge 0}$, $\boldsymbol{d}_{\lambda }^{w}\in \mathbb {Z}^{\mathcal {K}}_{\ge 0}$, and $\boldsymbol{d}_{\lambda }^{n}\in \mathbb {Z}^{\mathcal {K}}_{\ge 0}$ for all scenarios $\lambda \in \varLambda$, respectively, without further restrictions. In response to the new setting, we extend the rMDP and employ not only a single mask but multiple robust masks, ideally each feasible for a cluster of scenarios. To flexibly react to changes in demand, we aim at finding multiple robust masks that PCPs can switch between. For instance, if a PCP uses a mask that is tailored to low demand and notices an increase, they can switch to a mask designed for that level of demand. To facilitate the switch from a mask for lower demand to a mask for high demand, we require a nested construction as shown in the following definition. We use the notation $[n]:=\{1,\ldots ,n\}$.

### Definition 9

Masks $\mu ^1,\ldots ,\mu ^n$ are called *nested* if

1. they have the same number of chronic slots in every session $k\in \mathcal {K}$, i.e., $n_{\mu ^i}{(c,k)}=n_{\mu ^j}{(c,k)}$ for all i,j∈[n] and
2. mask $\mu ^j$ has at least as many acute slots as mask $\mu ^i$ for i<j in every session $k\in \mathcal {K}$, i.e., $n_{\mu ^i}{(a,k)}\le n_{\mu ^j}{(a,k)}$ for i<j with i,j∈[n].

With nested masks $\mu ^1,\ldots ,\mu ^n$, a PCP can always start using mask $\mu ^1$ and easily switch to another mask $\mu ^j$ with j>1. We note that the number of masks can be selected as required. However, if the number of masks is large, the masks will not differ significantly from each other but only by single slots. It is therefore advisable to adjust the number of masks to the fluctuation in demand. Given this new setting, we define a robust multimask.

### Definition 10

**(Robust Multimask)** A *multimask* $\boldsymbol{\mu }=(\mu ^1,\ldots ,\mu ^n)$ is defined by a tuple of nested masks $\mu ^i:\mathcal {S}\rightarrow \{c, a, w\}$, i∈[n]. It is called *robust* if a $(d^{c},\boldsymbol{d}_{\lambda }^{a})$-assignment exists for each scenario $\lambda \in \varLambda$ that is compatible with at least one of the masks of $\boldsymbol{\mu }$. The set of all $(d^{c},\boldsymbol{d}_{\lambda }^{a})$-assignments that are compatible with at least one mask of $\boldsymbol{\mu }$ is denoted by $\mathcal {A}^{\boldsymbol{\mu }}_\lambda$, $\lambda \in \varLambda$.

Before evaluating a robust multimask, we note that the PCP’s utilization $u^{}_{\lambda }$ depends now on the scenario $\lambda \in \varLambda$, i.e., $u^{}_{\lambda } =\frac{t}{C}\left( d^{c}+\sum \limits _{k\in \mathcal {K}}(d_{\lambda ,k}^{a} + d_{\lambda ,k}^{w}{- d_{\lambda ,k}^{w}})\right) .$

### Definition 11

Let the PCP’s capacity $\boldsymbol{c}\in \mathbb {Z}^{\mathcal {K}}_{\ge 0}$, demand $d^{c}\in \mathbb {Z}_{\ge 0}$, and demands $\boldsymbol{d}_{\lambda }^{a}, \boldsymbol{d}_{\lambda }^{w}, {\boldsymbol{d}_{\lambda }^{n}}\in \mathbb {Z}^{\mathcal {K}}_{\ge 0}$ for all scenarios $\lambda \in \varLambda$ be given. The *cost* of a robust multimask $\boldsymbol{\mu }$ is defined as

$$ c(\boldsymbol{\mu })= \max _{\lambda \in \varLambda } \min _{\begin{array}{c} \boldsymbol{\alpha }\in \mathcal {A}^{\boldsymbol{\mu }}_\lambda \end{array}} \max _{k\in \mathcal {K}} \left| {\varPhi _{\lambda ,k}^{\boldsymbol{\alpha }}} - u _{\lambda } \cdot c_{k} \right| . $$

Finally, we can define the rMMDP.

### Definition 12

**(rMMDP)** Let $\boldsymbol{c}\in \mathbb {Z}^{\mathcal {K}}_{\ge 0}$ be the PCP’s capacity, $d^{c}\in \mathbb {Z}_{\ge 0}$ be the chronic demand, and $\boldsymbol{d}_{\lambda }^{a}, \boldsymbol{d}_{\lambda }^{w}, {\boldsymbol{d}_{\lambda }^{n}}\in \mathbb {Z}^{\mathcal {K}}_{\ge 0}$ be the acute, walk-in, and no-show demands for all scenarios $\lambda \in \varLambda$, respectively. The *robust multimask design problem* aims at finding a robust multimask of minimum cost.

In the following, we also model the rMMDP by a network flow and design model. Therefore, let $\pi :\varLambda \rightarrow \{\mu ^1,\ldots , \mu ^n\}$ be a function that allocates one mask to each scenario. In network $N ^\varLambda$, a feasible *nested robust* $\boldsymbol{ \beta }$*-flow* $\boldsymbol{f}=(f^1,\ldots , f^{|\varLambda |})$ is defined by feasible $\beta ^\lambda$-flows $f^\lambda :A\rightarrow \mathbb {Z}_{\ge 0}$ for all scenarios $\lambda \in \varLambda$ that satisfy the flow properties presented in (F1)-(F3):

$$\begin{aligned} (F1) \hspace{0.25cm}&f^\lambda (a)=f^{\lambda ^\prime } (a) \text { for all }a\in A_{\text {fix}}^c\text { and }\lambda , \lambda ^\prime \in \varLambda , \\ (F2) \hspace{0.25cm}&f^\lambda (a)= f^{\lambda ^\prime }(a)\text { for all }a\in A_{\text {fix}}^a\text { and }\lambda , \lambda ^\prime \in \varLambda \nonumber \\&\text {with }\pi (\lambda )= \pi (\lambda ^\prime ),\\ (F3) \hspace{0.25cm}&f^\lambda (a)\le f^{\lambda ^\prime }(a)\text { for all }a\in A_{\text {fix}}^a\text { and } \lambda , \lambda ^\prime \in \varLambda \nonumber \\&\text {where }\lambda \in \pi ^{-1}(\mu ^i), \ \lambda ^\prime \in \pi ^{-1}(\mu ^j) \text { with }i\le j. \end{aligned}$$

Flow property (*F*1) ensures that the flow value on a fixed arc contained in set $A_{\text {fix}}^c$ is equal among all scenarios. Flow property (*F*2) ensures that the flow value on a fixed arc contained in set $A_{\text {fix}}^a$ is equal for all scenarios which are assigned to the same mask. Flow property (*F*3) ensures non-decreasing flow values on fixed arcs contained in set $A_{\text {fix}}^a$ among scenarios assigned to different masks. Analogous to the previous sections, a nested robust $\boldsymbol{ \beta }$-flow in network $N^{\varLambda }$ that minimizes the following expression

$$ \max _{\lambda \in \varLambda } \max _{k\in \mathcal {K}} | t\cdot f^{\lambda }(a^2_k) - u _{\lambda } \cdot c_{k} | $$

corresponds to a solution to the rMMDP. In contrast to the rMDP, the solution space of the rMMDP differs among the scenarios as the PCP’s utilization depends on the scenario considered. The possible solution values form the finite set $\varOmega ^{\varLambda } = \bigcup _{\lambda \in \varLambda ,k\in \mathcal {K}} \varOmega _{\lambda ,k}$ with

$$ \varOmega _{\lambda ,k}= \left\{ | t\cdot i - u _{\lambda } \cdot c_{k}| \Big \vert i\in \left\{ 0,1,\ldots , D_{\lambda ,k} \right\} \right\} , $$

where $D_{\lambda ,k} = d_{\lambda ,k}^{w} {-d_{\lambda ,k}^{n}}+ \sum _{k^\prime \in \mathcal {K}^{-}_{k}}d_{{\lambda ,k}^{\prime }}^{a} + d^{c}$. Analogous to the previous section, we use an adjusted version of Algorithm 1 to find the minimum parameter $\omega \in \varOmega ^\varLambda$ for which a feasible nested robust $\boldsymbol{ \beta }$-flow $\boldsymbol{f}$ exists in network $N ^{\varLambda }_\omega$. We determine a feasible nested robust flow using a compact IP formulation.

9 $$\begin{aligned} \min&\ \ {\sum _{\lambda \in \varLambda }\sum _{k\in \mathcal {K}}\sum _{( \sigma ^{a}_{k} , \sigma ^{w}_{k} )\in A}f_{a}^{\lambda } }  &   \end{aligned}$$

10 $$\begin{aligned}   [1em] \text { s.t. }&\sum _{a=(v,w)\in A} f_{a}^{\lambda } - \sum _{a=(w,v)\in A} f_{a}^{\lambda } = \beta ^{\lambda }(v)  &   \forall \ v \in V, \ \lambda \in \varLambda \end{aligned}$$

11 $$\begin{aligned}   [1em]&0\le f_{a}^{\lambda } \le \psi (a)  &   \forall \ a \in A, \ \lambda \in \varLambda \end{aligned}$$

12 $$\begin{aligned}   [1em]&{ \underline{\psi }_{\omega } (a)\le f_{a}^{\lambda } \le \overline{\psi }_{\omega } (a) }  &   \forall \ a=a_2^k \in A, \lambda \in \varLambda \end{aligned}$$

13 $$\begin{aligned}   [1em]&f_{a}^{\lambda } = f_{a}^{\lambda ^{\prime }}  &   \forall \ a \in A^{c}_{\text {fix}}, \ \lambda , \lambda ^{\prime } \in \varLambda \end{aligned}$$

14 $$\begin{aligned}   [1em]&\sum _{i=1}^{n}y^i_{\lambda } =1  &   \forall \ \lambda \in \varLambda \end{aligned}$$

15 $$\begin{aligned}   [1em]&f^\lambda _{a^1_k} \le m^i_{k} + n_k(1-y^i_{\lambda })  &   \forall \ i\in [n], \ k\in \mathcal {K}, \ \lambda \in \varLambda \end{aligned}$$

16 $$\begin{aligned}   [1em]&f^\lambda _{a^1_k} \ge m^i_{k} - n_k(1-y^i_{\lambda })  &   \forall \ i\in [n], \ k\in \mathcal {K}, \ \lambda \in \varLambda \end{aligned}$$

17 $$\begin{aligned}   [1em]&m^i_k\le m^{i+1}_k  &   \forall \ i\in [n-1], \ k\in \mathcal {K} \end{aligned}$$

18 $$\begin{aligned}   [1em]&f_{a}^{\lambda } \in \mathbb {Z}_{\ge 0 }  &   \forall \ a \in A, \ \lambda \in \varLambda \end{aligned}$$

19 $$\begin{aligned}   [1em]&m^i_k\in \mathbb {Z}_{\ge 0 }  &   \forall \ i\in [n], \ k\in \mathcal {K} \end{aligned}$$

20 $$\begin{aligned}   [1em]&y^i_{\lambda } \in \{0,1\}  &   \forall \ i\in [n], \ \lambda \in \varLambda \end{aligned}$$

Let $f_{a}^{\lambda }\in \mathbb {Z}_{\ge 0}$ be the variables that model the flow on arc a∈A in scenario $\lambda \in \varLambda$. Let $m^i_k\in \mathbb {Z}_{\ge 0}$, i∈[n] be auxiliary variables that indicate the amount of flow on arcs $a^1_k\in A$. Further, let $y^i_{\lambda }\in \{0,1\}$ be binary variables that determine whether a mask $\mu ^i$, i∈[n] is allocated to scenario $\lambda \in \varLambda$ ($y^i_{\lambda }=1$) or not ($y^i_{\lambda }=0$), corresponding to function π. The IP can be formulated as presented in Equations 9–20. Objective (9) minimizes the flow value that represents unsuccessful treatment requests. The flow balance constraints (10) and capacity constraints (11) ensure feasible flows in all scenarios. Capacity constraints (12) guarantee the desired balanced flow on the treatment arcs. Constraints (13) enforce the equal flow values on arcs $a\in A^{c}_{\text {fix}}$ among all scenarios as required in flow property (*F*1). Constraints (14) model that one mask is assigned to each scenario which corresponds to function π. Constraints (15) and (16) enforce the equal flow values on arcs $a \in A^{a}_{\text {fix}}$ (in amount of $m^i_k$) for two scenarios $\lambda , \lambda ^\prime \in \varLambda$ if the scenarios are assigned to the same mask, i.e., $\mu ^i= \pi (\lambda )= \pi (\lambda ^\prime )$, i∈[n] ($y^i_\lambda =y^i_{\lambda ^\prime }=1$), as required in flow property (*F*2). Otherwise, they function only as upper and lower bounds on the flows. The combination of constraints (15)–(17) enforce the non-decreasing flow values on arcs $a\in A^{a}_{\text {fix}}$ for two scenarios $\lambda , \lambda ^\prime \in \varLambda$ if the scenarios are assigned to different masks $\mu ^i$, $\mu ^j$ with i≤j, i.e., $\mu ^i= \pi (\lambda )$ and $\mu ^j= \pi (\lambda ^\prime )$ ($y^i_\lambda =y^j_{\lambda ^\prime }=1$), as required in flow property (*F*3). Finally, constraints (18)–(20) define the domain of the variables.

Overall, we obtain the following theorem.

### Theorem 3

A robust multimask determined by a nested robust $\boldsymbol{ \beta }$-flow obtained by the adjusted Algorithm 1 is of minimum cost.

### Proof

Analogous to the proof of Theorem 1.□

We refer to the extended model and procedure presented in this section as *RobMulti-F* and conclude with the following corollary.

### Corollary 3

RobMulti-F is a modeling for the rMMDP.

## Case study

In this section, we present a case study conducted to demonstrate the potential of the mask-based appointment scheduling systems obtained by solving MDP, rMDP, and rMMDP. In Section 6.1, we present the experimental environment for the case study. In Section 6.2, we present the setting of the implementation and computation. In Section 6.3, we discuss the results of the optimization models. In Section 6.4, we evaluate the mask-based appointment scheduling systems and compare them with some appointment scheduling systems from the literature using agent-based simulation.

### Experimental environment

In this section, we present the experimental environment for the case study. This includes the physician-patients setting, the simulation model and its input data, and the appointment scheduling systems. In addition, this includes the instance generation and input parameter for the optimization models. We note that our setting is tailored to a German primary health care scenario on the basis of our knowledge and available data. We use this scenario to show the potential of the mask-based system. Naturally, the mask-based appointment scheduling systems can be tailored to different scenarios and settings as required.

**Table 2:** Patient configurations

Config.	# patients	chronic. ill	walk-in	no-show
ID		(%)	(%)	(%)
1	1400	40	15	10
2	1400	40	18	10
3	1400	40	20	10
4	1400	45	15	10
5	1400	45	18	10
6	1400	45	20	10
7	1600	40	15	10
8	1600	40	18	10
9	1600	40	20	10
10	1600	45	15	10
11	1600	45	18	10
12	1600	45	20	10
13	2000	40	15	10
14	2000	40	18	10
15	2000	40	20	10
16	2000	45	15	10
17	2000	45	18	10
18	2000	45	20	10

#### Physician-patients setting

In primary health care, the physician-to-patient ratio is commonly used to measure medical care. Following the German Federal Joint Committee’s (GFJC) guideline [49], we set the ratio of one PCP per 1607 patients as a benchmark for analyzing the appointment scheduling systems in this study. The GFJC defines an excess of 10% in the ratio as oversupply, i.e., one PCP per 1448 patients. An undersupply is defined by a 25% shortfall in the ratio, i.e., one PCP per 2011 patients. Representing these three scenarios, we analyze the performance of the appointment scheduling systems for populations of 1400, 1600, and 2000 patients. According to a study of the German Robert Koch Institute [50], 43% of female and 38% of male patients are chronically ill. As our study is gender neutral, we assume that 40% of all patients suffer from chronic illnesses. Due to the worldwide aging population with the highest risk of chronic conditions, an increase in the number of chronically ill patients is expected. Thus, we analyze a chronic proportion of 40% and 45%. Over the past ten years, surveys conducted by the National Association of Statutory Health Insurance Physicians (NASHIP) [19] have revealed that on average 18.8% of the patients holding health insurance are walk-ins that forgo scheduling an appointment with their PCPs. To cover this and slight deviations, we analyze a walk-in rate of 15%, 18%, and 20%. Furthermore, the NASHIP found that the no-show rates of 86% of German primary care practices are only up to 10% [19]. Therefore, it is not surprising that 83% of practices consider no-shows to be no problem, only a very minor problem, or a minor problem. Based on these results, we consider a uniform no-show rate of 10%. In total, we obtain 18 different configurations for the patient population considered in this case study, as summarized in Table 2.

Based on a survey regarding the practice’s opening hours conducted by The National Association of Statutory Health Insurance Funds [51], we determine the PCP’s opening hours as follows. We decide the PCP to operate in a four-hour morning session from Monday to Friday and in a two-and-a-half-hour afternoon session on Mondays, Tuesdays, and Thursdays. This results in a time span $o_{(i,0)}=240$ for $i\in \{0,\ldots ,4\}$ and $o_{(i,1)} =150$ for $i\in \{0,1,3\}$. The first hour after each session is used as buffer time, i.e., $b_{k}=60$ for all $k\in \mathcal {K}$. Accordingly, overtime is caused if the PCP works beyond the capacity $c_{(i,0)}=300$ for $i\in \{0,\ldots ,4\}$ and $c_{(i,1)}=210$ for $i\in \{0,1,3\}$.

#### Simulation model SiM-Care and modifications

To generate instances and to analyze the appointment scheduling systems designed in this study, we use the agent-based simulation tool ’SiM-Care’ (Simulation Model for Primary Care) [52]. SiM-Care simulates the dynamics of primary health care systems and aims at supporting decision-makers in their planning, analysis, and adaption. For this purpose, it models PCPs, patients, and their interaction on an individual level. Tracking the interactions allows identifying the interdependencies between different subproblems, evaluating new planning approaches, and quantifying the effects of interventions based on multiple performance measurements. SiM-Care enables in particular the comparison and assessment of appointment scheduling systems in terms of, for example, patients’ waiting times, patients’ access times, or the PCPs’ utilization.

**Table 3:** Basis for the selection of input parameters

Aspects	Distribution
frequency of acute illnesses	exponential distribution
type of acute illnesses	age-class-illness distribution
seriousness of acute illnesses	triangular distribution
duration of acute illnesses	log-normal distribution
patients’ willingness to wait	Weibull distribution
patient punctuality	normal distribution
walk-in arrivals	beta distribution
service time	log-normal distribution
appointment cancellations	binomial distribution
appointment/walk-in	discrete probability distr.
show/no-show	discrete probability distr.

SiM-Care relies on stochastic values to approximate real-world variability and control the frequency of events. This applies to aspects of illnesses as well as to patient arrivals, appointment cancellations, and service times. Table 3 lists all probabilistic model aspects of SiM-Care. We refer to the study of Comis et al. [52] for details of the parameterization of the distributions underlying the random values. For the sake of validity, SiM-Care uses real-world data to the extent available. For example, the distribution of illnesses and their characteristics are estimated on the basis of publications of health insurances and federal government agencies. All unavailable data was either empirically collected in a primary care practice, for instance the patients’ service times, or where this was not possible, it was inferred as described in more detail in the next section.

To simulate the needs of our primary health care setting, we make the following changes to SiM-Care. First, PCPs may reject patients due to capacity overloads in the current modeling. Rejected patients then seek care at other PCPs. As we only consider one PCP in our study, we integrate an emergency practice to SiM-Care. Accordingly, if patients are rejected by the PCP in our setting, they seek immediate care at a physician in the emergency practice. Second, patients always try to make an appointment first in the current modeling. If they succeed, they arrange appointments, which they later attend or cancel. Otherwise, they seek immediate care as walk-in within their willingness to wait. We note that the patients’ willingness to wait in SiM-Care follows a Weibull distribution and depends on factors such as the illness, seriousness of the illness (depending on the patient’s health condition) with the resulting expected willingness to wait, and the deviation from the expected willingness to wait due to patient’s age. We integrate the feature that patients forgo making an appointment and instead seek immediate care as walk-in. Furthermore, we integrate the feature that patients do not show up to their arranged appointments. The percentages of walk-ins and no-shows can be defined by the user. Third, we assume that walk-ins are subject to the same service time log-normal distribution as patients with appointments.

Before addressing the input parameters for SiM-Care in the next section, we note that SiM-Care is a complex simulation model, much more complex than our optimization models. We have chosen SiM-Care on an operational decision level to prove that the optimization models developed on a tactical decision level can withstand complex systems.

#### Input parameters for SiM-Care

Comis et al. [52] present a case study for a primary health care system in Germany to showcase SiM-Care and its validation through expert input and empirical data. Specifically, they create a baseline scenario representing a real-world primary health care system. In this study, we conduct a case study based on this baseline scenario. In the following, we detail the most important input parameters and the parameter choices that deviate from the baseline scenario. Table 4 summarizes the selection of input parameters. For further details, we refer to the study of Comis et al. [52].

**Table 4:** Basis for the selection of input parameters

Attribute	Basis (Source)
PCP’s	
opening hours	empirical ([51])
strategies	literature ([52–54])
Patients’	
age class	empirical ([55])
health condition	inferred ([52])
service times	empirical ([52])
Age classes	
exp. annual acute illnesses	inferred ([52])
dev. illness duration	inferred ([52])
dev. willingness to wait	inferred ([52])
availabilities	inferred ([52])
appointment cancellations	inferred ([52])
chronic patients	empirical ([50])
Families of illnesses	
characteristics	inferred ([52])
age-class illness distribution	empirical ([56])

First, we model a time period of one year (52 weeks) preceded by a warm-up period of also one year. Second, we consider randomly generated locations of the patients and physicians as performance indicators such as travel times are not important in our case study. Third, the modeled PCP and their opening hours as well as the modeled patients are as described in Section 6.1.1. Fourth, we assume the same distributions of age classes and health conditions of patients as in the baseline scenario. This includes the estimations of the data of age-class-dependent attributes for each patient as listed in Table 4. Fifth, we consider the same families of illnesses as in the baseline scenario, consisting of three chronic and four acute illnesses as listed in Tables 5 and 6, which is a subset of the 100 ICD-10 codes most frequently reported to the Association of Statutory Health Insurance Physicians Nordrhein [57].

**Table 5:** Chronic illness families $f\in \mathcal {F}$ with respective expected willingness to wait (WTW) depending on seriousness s∈[0,1]

ICD	Name	exp. WTW $W_f$
I10	high blood pressure	$W_f(s)=-10s+20$
E11	diabetes	$W_f(s)=-4s+14$
I25	ischemic heart disease	$W_f(s)=-4s+10$

Sixth, we apply the same estimations for the characteristics of the families of illnesses such as expected willingness to wait (cf. Tables 5 and 6), duration or the treatment frequency, as real data is protected by confidentiality and not published. The only difference is that we assume that patients with acute illnesses do not require a follow-up treatment (i.e., the treatment frequency is not applicable). Seventh, we consider the same data for the age-class illness distribution estimated on the basis of the reported incidence rates of a large German health insurer [56]. Finally, we present the integrated appointment scheduling, the treatment, and the admission strategy employed by the PCP modeled in SiM-Care. The integrated appointment scheduling strategies are detailed in Section 6.1.4. The treatment strategy is defined as in the baseline scenario [52]. The strategy includes the order of treatments on a priority-first-come-first-served basis: Patients with appointment are prioritized over walk-ins and within their respective groups, patients are served in order of their arrival, i.e., first come, first served. In addition, the treatment strategy includes the PCP’s consultation speed, that is, the PCP adjusts the service times whenever more than three patients await treatment. The service times are sampled from a maximum likelihood fitted log-normal distribution based on empirical service times resulting in a mean service time of 7.8 minutes. The admission strategy is defined as follows. Like in studies [53, 54], walk-ins are admitted up to a certain utilization threshold. Therefore, PCPs predict their remaining workload by multiplying the average service time of 7.8 minutes with the number of currently waiting patients and upcoming scheduled appointments. If this estimated workload is lower than the remaining duration of the current session including buffer time, walk-ins are admitted, otherwise rejected. Patients with appointments are admitted as long as they arrive before the end of the buffer time. We note that patients with appointments do not always arrive on time in SiM-Care. Patient arrivals deviate around the scheduled time of the appointment and follows a normal distribution with a mean arrival deviation of -5 minutes and standard deviation of 6 minutes such that roughly 20% of all patients are likely to arrive late for their appointments.

**Table 6:** Acute illness families $f\in \mathcal {F}$ with respective expected willingness to wait (WTW) depending on seriousness s∈[0,1]

ICD	Name	exp. WTW $W_f$
E78	high cholesterol level	$W_f(s)=-5s+8$
M54	back pain	$W_f(s)=-3s+4$
Z25	vaccination	$W_f(s)=40$
J06	cold	$W_f(s)=-2s+2$

**Fig. 4:**
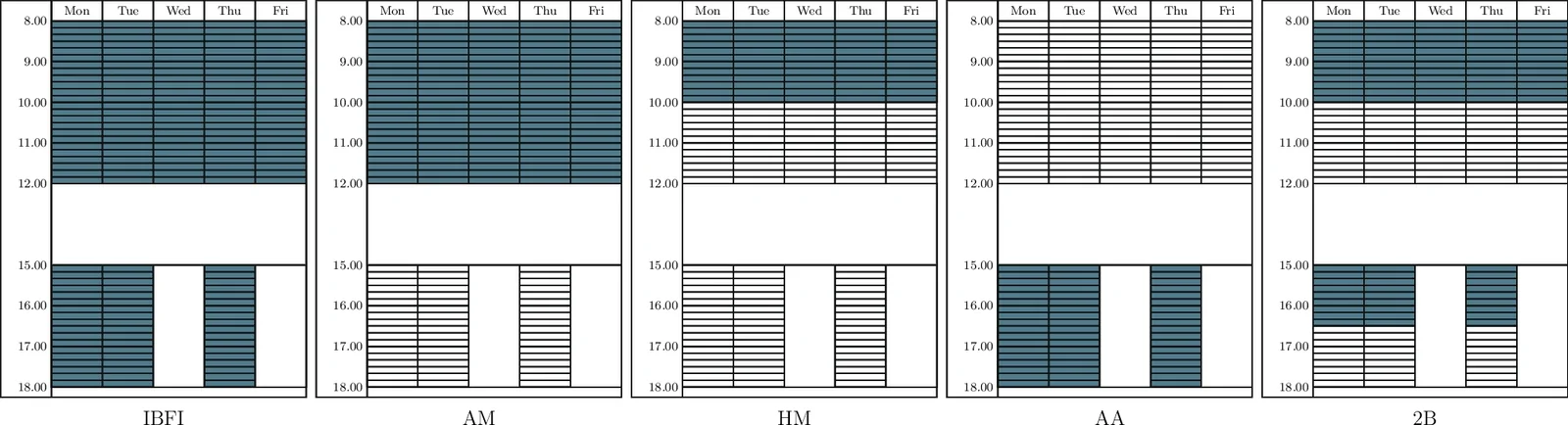
Non-mask-based systems from the literature with slots  reserved for appointments and slots  withheld for walk-ins

#### Appointment scheduling systems

In this section, we present the PCP’s appointment scheduling strategies, or in other words, the appointment scheduling systems that we implement in SiM-Care for this study. Before presenting the considered appointment scheduling systems, we note that they are based on slot systems. To that end, the opening hours of each session are divided into slots of 10 minutes length. As the NASHIP [19] reveals that only 22% of German practices respond to no-shows with overbooking, we do not implement an overbooking strategy. This means that theoretically each slot can accommodate one appointment. However, the appointment scheduling system determines which slots are allowed to accommodate an appointment. In SiM-Care, appointments are offered to patients on a first-come-first-serve basis. Consequently, every patient is offered the earliest feasible appointment at the time of inquiry, i.e., immediately after the onset of illness. If no feasible appointment is available, the patient decides to forgo an appointment and seek immediate care as walk-in. An offered appointment is feasible for a patient if it matches with the patient’s availabilities and if it is within the patient’s willingness to wait. We note that acute patients may cancel their appointments if they recover before. In the following, we list the implemented appointment scheduling systems. We refer to Fig. 4 which visualizes the five appointment scheduling systems derived from the literature.

##### Individual-Block/Fixed-Interval (IBFI)

Like Comis et al. [52], we consider the IBFI appointment scheduling system. IBFI allows all slots of each session to accommodate an appointment [24, 58]. Thus, no slots are withheld.

##### All Morning (AM) and Half Morning (HM)

Like Balasubramanian et al. [5] and Wiesche et al. [20], we consider the AM and the HM appointment scheduling system as benchmarks. AM allows all slots of each morning session to accommodate an appointment. HM allows the first half of the slots of each morning session to accommodate an appointment. In both appointment scheduling systems, the slots of every afternoon session are withheld for treatments of walk-ins.

##### All Afternoon (AA)

Like Balasubramanian et al. [5], we consider the AA appointment scheduling system as a benchmark. AA allows all slots of each afternoon session to accommodate an appointment. The slots of every morning session are withheld for treatments of walk-ins.

##### 2-Block (2B)

We consider the 2B appointment scheduling system introduced by Balasubramanian et al. [5]. 2B allows the slots of the first half of each session to accommodate an appointment. The slots of the second half of each session are withheld for treatments of walk-ins.

##### Masked-Based (M) and Robust-Mask-Based (RM)

We consider the M and the RM appointment scheduling systems that are based on a mask and a robust mask obtained by solving MDP and rMDP, respectively. M and RM allow in each session the chronic and acute slots to accommodate an appointment for one chronic or acute patient, respectively. The walk-in slots of each session are withheld for treatments of walk-ins.

##### Multimask-Based (MM)

We consider the MM appointment scheduling system based on a multimask consisting of three nested masks obtained by solving rMMDP. MM allows in each session the chronic and acute slots to accommodate an appointment for one chronic or acute patient, respectively. Every acute patient is offered the earliest feasible appointment of the first mask at the time of inquiry. If the earliest feasible appointment is not within the patient’s willingness to wait, the patient is offered the earliest feasible appointment of the second and if necessary of the third mask. The walk-in slots of each session are withheld for treatments of walk-ins.

We refer to AM, HM, and AA as benchmark systems, to IBFI, AM, HM, AA, 2B as non-mask-based systems, and to M, RM, and MM as mask-based systems. Before we conclude this section, we note that the non-mask-based systems do not distinguish between slots reserved explicitly for acute or chronic patients. To make the comparison between the non-mask-based and mask-based systems fairer, we implement slots reserved for chronic patients in the non-mask-based systems in amount of the average weekly chronic demand $d^{c}$. The chronic slots are evenly distributed among the sessions with slots that allow to accommodate an appointment. All remaining slots that allow to accommodate an appointment are reserved for acute patients. In the AM system, for example, we distribute the chronic slots across all morning sessions. The remaining slots of the morning sessions are reserved for acute patients and the slots of every afternoon session are still withheld for treatments of walk-ins. This adjustment means that all appointment scheduling systems considered in this study offer the same number of chronic slots per week.

#### Instance generation and input parameters for (robust) mask and robust multimask design problem

For the instances of MDP, rMDP, and rMMDP, demand data is crucial. In the absence of accessible empirical data on visits to primary care practices, we have to rely on simulation to obtain rough estimates. Based on a selected patient configuration presented in Section 6.1.1, we use SiM-Care [52] to simulate the number of visits to a primary care practice by acute and chronic patients as well as the numbers of walk-ins and no-shows over a one-year time horizon. We obtain the demand of 52 weeks and classify it into acute, chronic, walk-in, and no-show demand. We prepare this demand data as input to our optimization problems as explained in the following.

For the MDP, we average the demand of the 52 weeks and obtain the session-specific acute, walk-in, and no-show demands $\boldsymbol{d}^{a}, \boldsymbol{d}^{w}, \boldsymbol{d}^{n}$ and the weekly chronic demand $d^{c}$. For the rMDP and the rMMDP, we require different scenarios, where each scenario consists of one week’s demand. For the input of both problems, we use the scenario set $\varLambda ^{52}=\{\lambda _1,\ldots ,\lambda _{52}\}$ that is obtained by considering the demand from each of the 52 weeks of the year as individual scenarios. As shown in the next section, the model RobMulti-F cannot optimally solve all instances to the rMMDP within the given time limit. In addition, we note that there is a risk of overfitting for the robust model Rob-F if the scenario set is too large due to the strict equal flow property (*F*). The same holds true for RobMulti-F due to the strict equal flow properties (*F*1) and (*F*2). For this reason, we use a second scenario set $\varLambda ^{13}$ as input for the rMDP and the rMMDP. Scenario set $\varLambda ^{13}=\{\lambda _1,\ldots ,\lambda _{13}\}$ is constructed by aggregating the demand of each four consecutive weeks of the 52 weeks to create 13 different scenarios. For the two scenario sets $\varLambda ^{52}$ and $\varLambda ^{13}$, we perform the following additional processing to obtain valid inputs to the optimization problems. For the rMDP, we standardized demand so that the total acute, walk-in, and no-show demands are equal in all scenarios, i.e., $\sum _{k\in \mathcal {K}} d_{\lambda ,k}^{a} = D^a$, $\sum _{k\in \mathcal {K}} d_{\lambda ,k}^{w} = D^w$, and $\sum _{k\in \mathcal {K}} d_{\lambda ,k}^{n} = D^n$ for all $\lambda \in \varLambda ^{52}$, $\lambda \in \varLambda ^{13}$. Furthermore, we standardize the chronic demand as it is specified by only one parameter $d^{c}$ in rMDP. For the rMMDP, we only standardize the chronic demand.

Since SiM-Care relies on stochastic values, our case study includes ten independent runs. Therefore, we perform the demand generation and processing described above ten times for each patient configuration. Given the ten runs for the 18 different patient configurations, we obtain a total of 180 instances for Det-F and, due to the two different scenario sets considered, 360 instances for Rob-F and RobMulti-F. Besides the demand data, the optimization problems MDP, rMDP, and rMMDP require the following input parameters. For each of the three optimization problems, we set the uniform anticipated service time t according to the mean service time in SiM-Care, which averages 7.8 minutes. Adjusted to the anticipated service time, we determine ten-minutes slots, i.e., ℓ=10. The capacity and buffer time of the PCP is set as described in Section 6.1.1. For the robust model presented in Section 5, we consider multimasks that consist of three nested masks, i.e., n=3, to generate a mask for weeks with low, medium, and high demand.

**Fig. 5:**
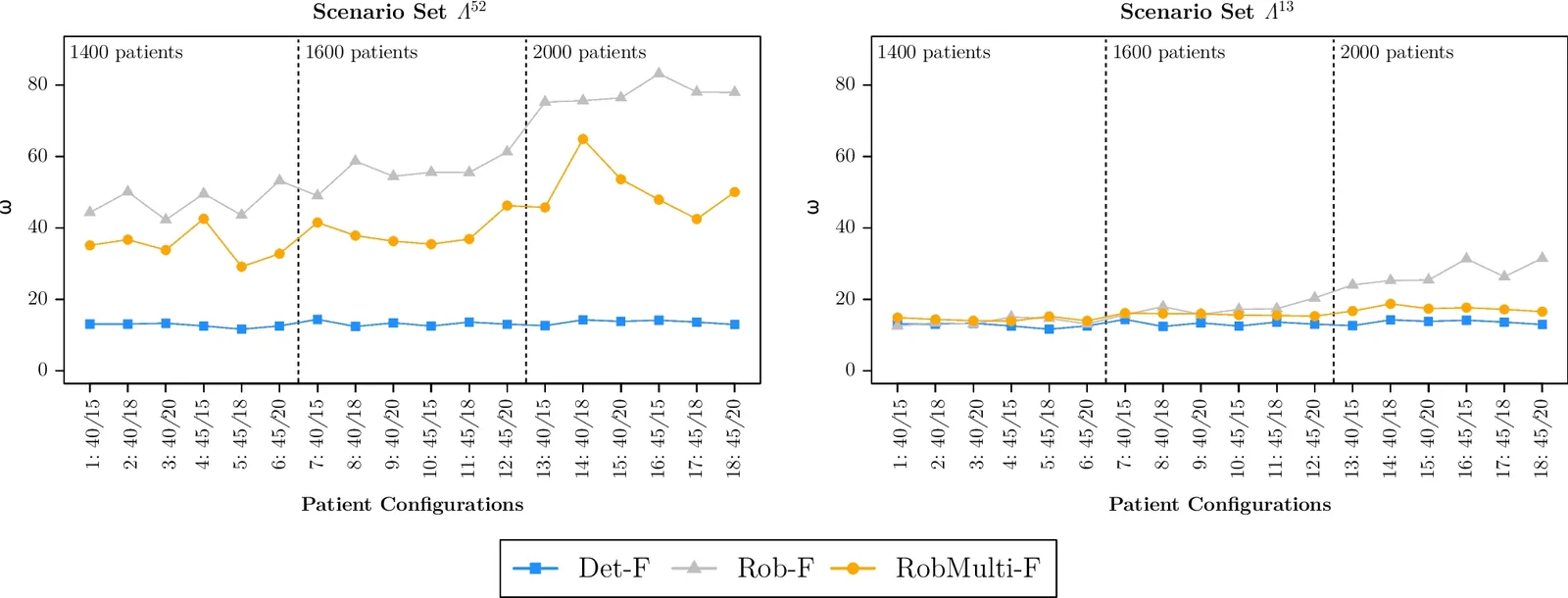
Average cost of masks ω depending on the scenario set ($\varLambda ^{52}$, $\varLambda ^{13}$), patient configuration (ID: chronic/walk-in), and optimization model (Det-F, Rob-F, RobMulti-F)

### Implementation and computational setting

For our computational study, all computational experiments are performed on a cluster of machines running Ubuntu 20.04.6 with an Intel(R) Core(TM) i9-9900 CPU @ 3.10 GHz and 32 GB DDR4-Non-ECC main memory. We restrict each individual job to one physical core, 14 GB main memory, and a time of 24 hours. We implement our algorithms based on binary search and IP formulations in Java using OpenJDK 11 [59]. To solve the IP formulations, we use CPLEX 22.1 through the Java API [60]. We restrict the CPLEX optimizer to an one hour time limit and 32 GB memory limit and leave all other CPLEX parameters at their default settings.

We solve the MDP, the rMDP, and the rMMDP by using the models Det-F, Rob-F, and RobMulti-F, respectively. Each model includes two optimization stages as presented in Sections 4.1, 4.2, and 5. In theory, we can solve both stages at once by determining flows of minimum cost instead of feasible flows. In contrast, when solving the models, we consider the stages separately and sequentially to speed up the computations. In the first optimization stage, we aim at finding the minimum parameter $\omega \in \varOmega$ for which a feasible flow in network $N_\omega$ exists by binary search. Once the minimum parameter $\omega \in \varOmega$ is found, we proceed with the second optimization stage. In the second optimization stage, we aim at finding a minimum cost flow, robust flow, or robust nested flow in network $N_\omega$. We recall that there does not exist a polynomial-time algorithm to determine a robust or a robust nested flow. Therefore and for simplicity’s sake, we compute all flows of all models, including the deterministic model, by IPs. While runtime is clearly not a focus of this study, we note the following regarding the solvability. The first optimization stage is performed in a few seconds for most instances and in less than three and a half hours for every instance. Consequently, for all instances an optimal solution to the first stage and thus a feasible solution to the second stage is found. The second optimization stage can be solved to optimality (CPLEX default MIP gap tolerance $10^{-4}$) for 66 out of the 180 instances based on scenario set $\varLambda ^{52}$ within the given time limit of one hour. For the remaining 114 instances, the best solution found so far is considered. As described in Section 6.1.5, due to non-solvability within the given time limit, we additionally consider instances based on scenario set $\varLambda ^{13}$ as input for the optimization problems Rob-F and RobMulti-F. For scenario set $\varLambda ^{13}$, all instances are solved to optimality within the given time limit.

**Fig. 6:**
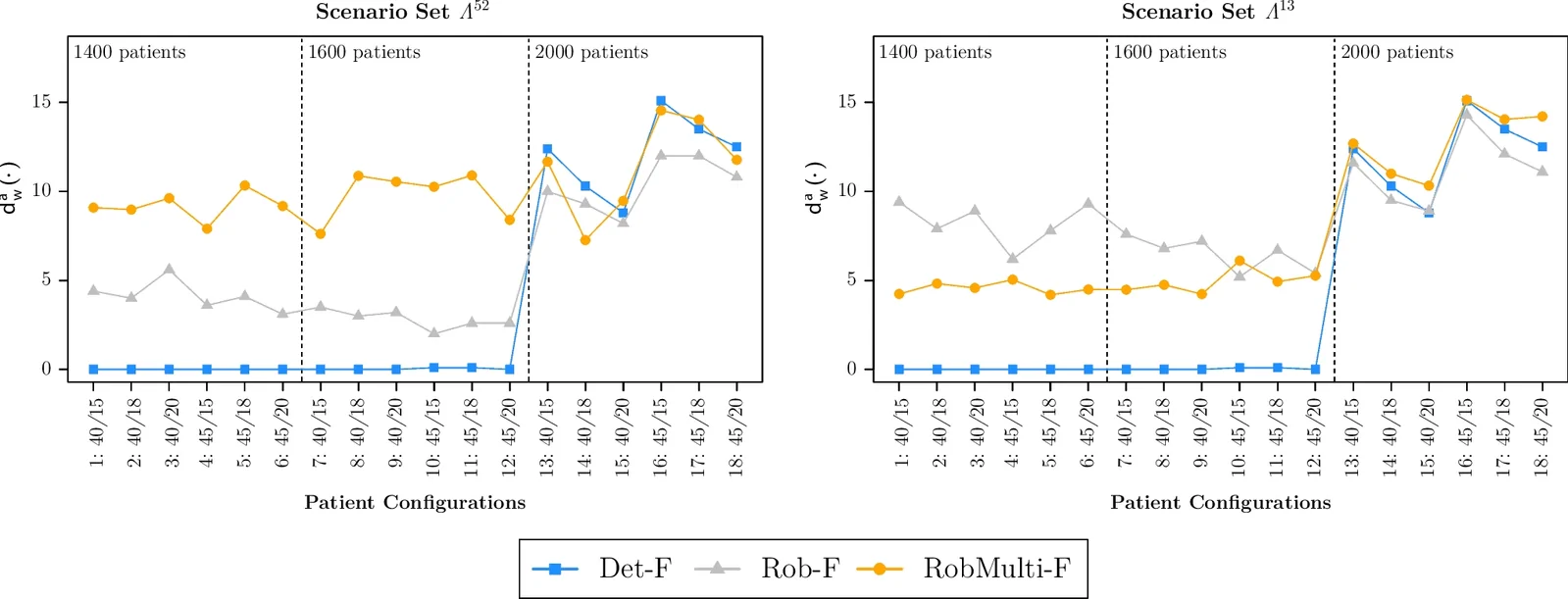
Average number of unsuccessful appointment requests $d^{a}_{\omega }(\cdot )$ depending on scenario set ($\varLambda ^{52}$, $\varLambda ^{13}$), patient configuration (ID: chronic/walk-in), and optimization model (Det-F, Rob-F, RobMulti-F)

### Evaluation of the optimization models and the resulting masks

In this section, we analyze the results of the Det-F, the Rob-F, and the RobMulti-F model. Before we begin, we recall the following. The solution of each model provides us with the following three results. First, we obtain the objective value $\omega \in \mathbb {Z}_{\ge 0}$ for which the respective flow with minimum cost is found in the corresponding network. Second, we obtain the number of the unsuccessful appointment requests $\left( d_{w}^{a}\bigl (\cdot \bigr )\right)$ by the cost of the flow. Third, we obtain a mask (of cost ω) and thus the number of chronic, acute, and walk-in slots $\left( \sum _{k\in \mathcal {K}}n_{\mu }\bigl (\cdot ,k \bigr )\right)$ by the flow itself. For all instances, the average results of the three models are summarized in Tables 7–8 in Appendix B.

We start by considering the objective value ω. For each model, the average objective value of the ten runs is visualized for all patient configurations and both scenario sets in Fig. 5. We recall that the results of Det-F are not computed based on the demands of the scenario sets $\varLambda ^{52}$ and $\varLambda ^{13}$, instead the input is the average annual demand. The double visualization is only for the comparability of the models. For Rob-F and RobMulti-F, the average objective value ω is affected by the choice of the scenario set. We confirm that the results for scenario set $\varLambda ^{52}$ are significantly higher than those for scenario set $\varLambda ^{13}$. This meets our expectations as the computation of the minimum parameter ω becomes more difficult the more scenarios are considered in the sense that the parameter must be suitable for more scenarios. Accordingly, Det-F achieves the best average objective values since the minimum parameter ω is determined for only a single demand scenario. We confirm that the patient configuration has no influence on these results. Considering the fact that the objective value ω corresponds to the cost of an optimal mask, we have to pay on average higher cost of a robust or robust nested mask compared to a non-robust mask, the so-called price of robustness [61]. RobMulti-F achieves for both scenario sets on average better objective values than Rob-F. This also meets our expectation due to the greater flexibility of a robust nested multimask compared to a robust mask. Furthermore, Rob-F and RobMulti-F have the tendency that the average objective value ω increases (with exceptions) the more patients are considered (patient configurations 1-6 versus 7-12 versus 13-18). Finally, recall that parameter ω or in other words the cost of a corresponding mask indicates the deviation of the PCP’s actual workload from the optimal workload using the respective model.

In the next step, we consider the total number of unsuccessful appointment requests $\left( d_{w}^{a}\bigl (\cdot \bigr )\right)$. For each model, we average the total number of unsuccessful appointment requests over the number of scenarios to obtain the average number per scenario. We visualize the average of the ten runs for all patient configurations and both scenario sets in Fig. 6. Det-F satisfies all appointment requests for patient configurations 1-6 and misses only a maximum average of 0.1 requests for patient configurations 7-12. In contrast, it performs significantly worse for the patient configurations 13-18 based on the population of 2000 patients with an average of 8.8 to 15.1 unsuccessful appointment requests. For Rob-F and RobMulti-F, the average number of unsuccessful appointment requests is affected by the choice of the scenario set. For patient configurations 1-12, the results of Rob-F are clearly lower than those of RobMulti-F for scenario set $\varLambda ^{52}$ while almost the opposite is the case for scenario set $\varLambda ^{13}$. We explain this by the following two reasons. First, as RobMulti-F is not optimally solved for all instances based on scenario set $\varLambda ^{52}$ (unlike set $\varLambda ^{13}$), only the best results found so far are considered for the total number of unsuccessful treatment requests. Second, the results of the objective values ω of Rob-F (cf. Fig. 5) are significantly higher for instances based on scenario set $\varLambda ^{52}$ than set $\varLambda ^{13}$. Consequently, the solutions to Rob-F based on scenario set $\varLambda ^{52}$ allow a higher deviation of the actual from the optimal PCP’s workload which in turn provides more flexibility for appointments so that there are fewer unsuccessful appointment requests. Like Det-F, Rob-F and RobMulti-F perform for patient configurations 13-18 significantly worse than for all other configurations. Consistent with the results, we expect that if the total number of patients increases while the PCP’s capacity remains the same, the number of unsuccessful appointment requests increases. A second result we expect is that if the walk-in rate increases, the number of patients that request an appointment decreases. Consequently, the number of unsuccessful appointment requests also decreases. However, this effect can only be observed in the results of all three models for configurations 13-18. We conclude with the following note. The number of unsuccessful appointment requests provides us with the information that, given an allowed maximum deviation, there is theoretically no assignment of the patient demand that would satisfy every appointment requests.

**Fig. 7:**
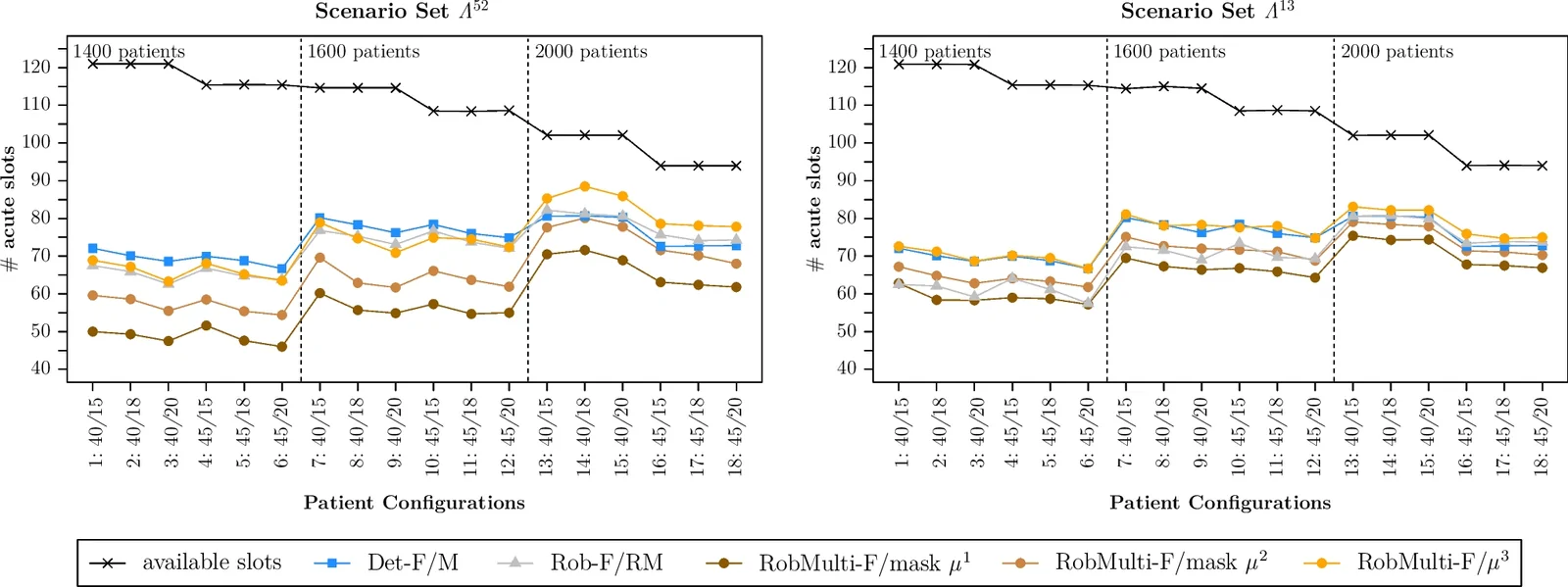
Average number of acute slots of the masks depending on the scenario set ($\varLambda ^{52}$, $\varLambda ^{13}$), patient configuration (ID: chronic/walk-in), and optimization model (Det-F, Rob-F, RobMulti-F)

Finally, we consider the structure of the masks, robust masks, and nested robust masks that result from the optimization models Det-F, Rob-F, and RobMulti-F, respectively. Before discussing the results, we note that there are in total $|\mathcal {S}|=165$ slots within the PCP’s capacity. Furthermore, we note that in all three models, $d^{c}$ out of the 165 slots are assigned the state ‘chronic’. Consequently, the number of available slots for state ‘acute’ or ‘walk-in’ is $165 -d^{c}$. In the following, we only compare the number of acute slots of the masks among all models. For each model, the total number of acute slots is averaged over the ten runs and visualized for all patient configurations and both scenario sets in Fig. 7.

First of all, we confirm that the number of slots available for state ‘acute’ decreases if the population of patients and thus the proportion of chronic patients increases. Moreover, we note that none of the models assigns all available slots to the state ‘acute’. Conversely, in all models there are slots assigned the state ‘walk-in’. For Det-F, the average total number of acute slots is at a high level compared to the results of the other models and varies between 66.7 and 80.7, depending on the patient configuration. For Rob-F and RobMulti-F, the total numbers of acute slots are affected by the choice of the scenario set. Before discussing the differences, we confirm for RobMulti-F the resulting nested masks, denoted by $\mu ^1$, $\mu ^2$, $\mu ^3$. Considering Rob-F, we see that the total number of acute slots is higher for the robust mask based on scenario set $\varLambda ^{52}$ than set $\varLambda ^{13}$. For RobMulti-F based on scenario set $\varLambda ^{52}$, we note that the total number of acute slots of the third mask is lower than those from Det-F neglecting patient configurations 13-18. In contrast, for RobMulti-F based on scenario set $\varLambda ^{13}$, the results almost coincide with those from Det-F. Moreover, we note that the difference of the number of acute slots between the three resulting masks is smaller for RobMulti-F based on scenario set $\varLambda ^{13}$ than on set $\varLambda ^{52}$. We explain this by the overall fewer and less diverse demand scenarios of set $\varLambda ^{13}$ used for the computation of the solution due to the aggregation of the scenarios. Furthermore, we see that each of the three masks based on scenario set $\varLambda ^{13}$ offer more acute slots than each of the corresponding masks based on set $\varLambda ^{52}$. All three models have the tendency that the total number of acute slots decreases if the walk-in rate increases as seen in almost all patient configurations, namely 1-3, 4-6, 7-9, 10-12, and 16-18.

**Fig. 8:**
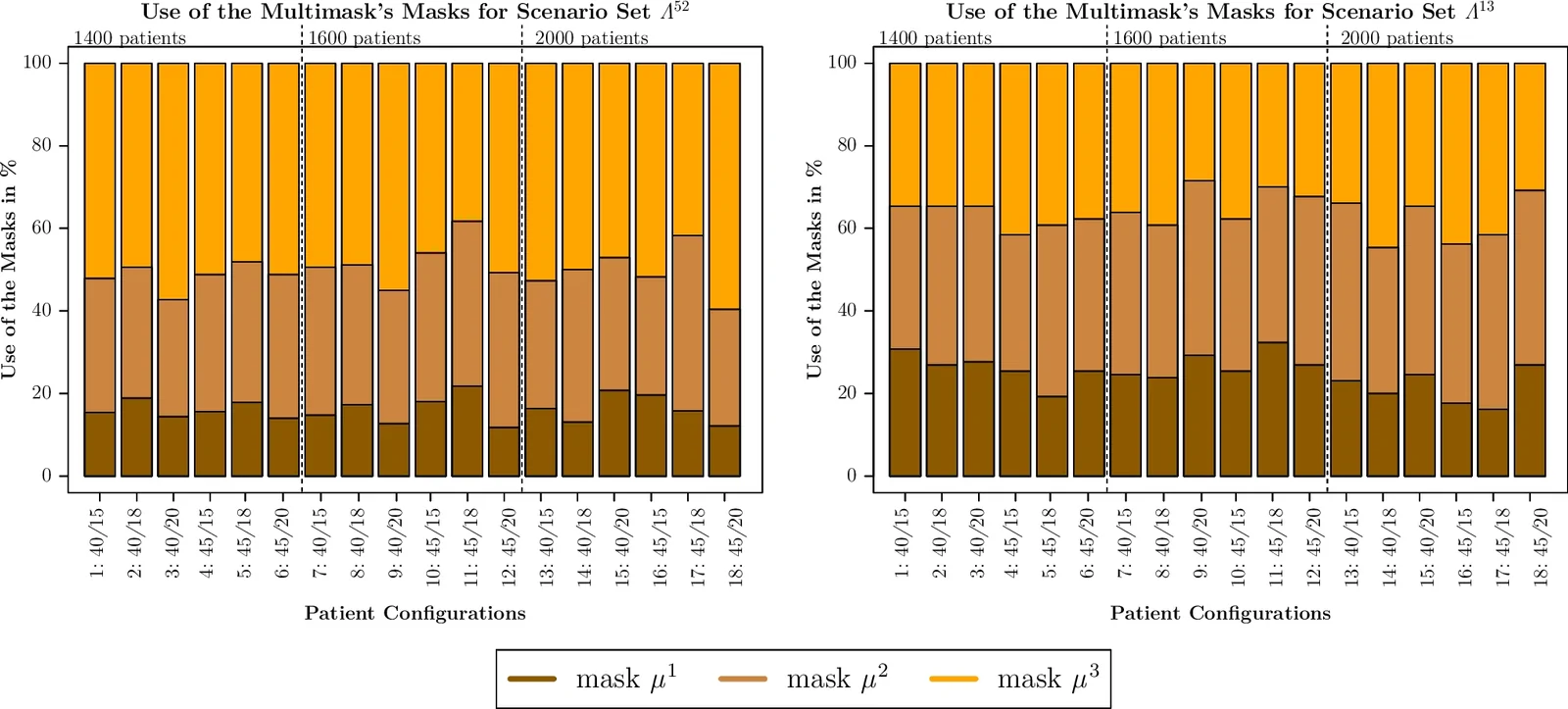
Percentage use of masks $\mu ^1$, $\mu ^2$, and $\mu ^3$ depending on the scenario set ($\varLambda ^{52}$, $\varLambda ^{13}$) and patient configuration (ID: chronic/walk-in)

**Fig. 9:**
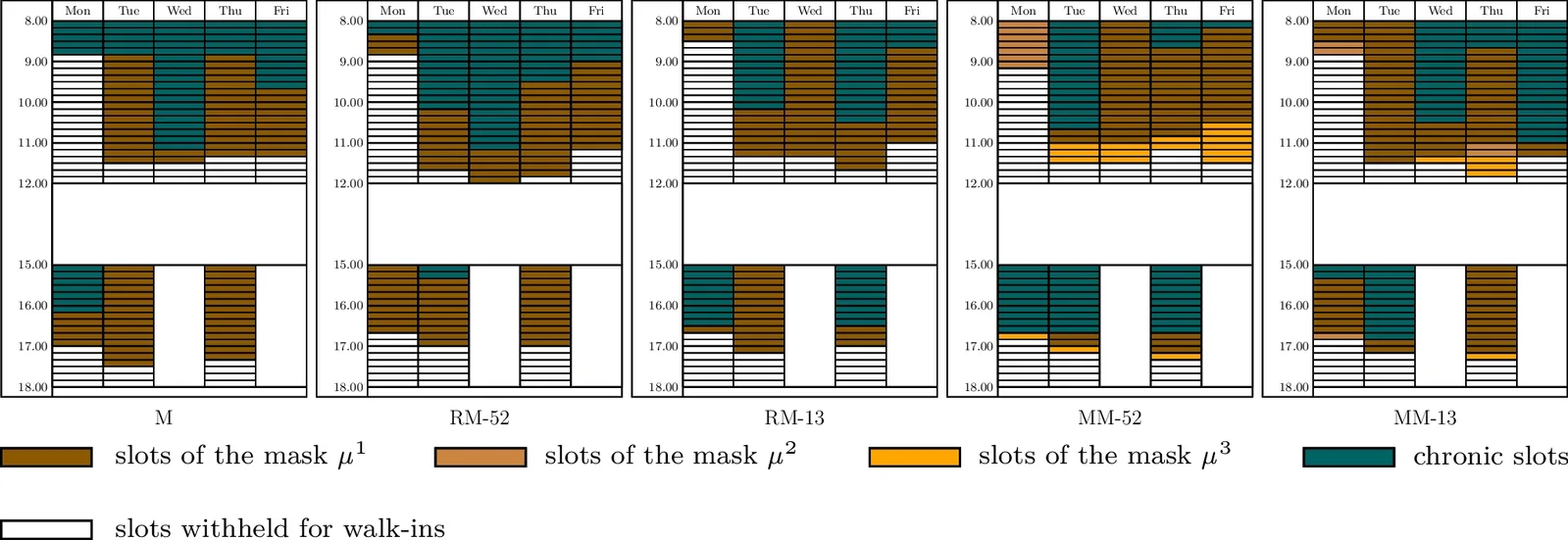
The resulting mask, robust mask, and robust nested mask of the tenth run for patient configuration 8

Before concluding the section, we analyze how often each of the three resulting masks of RobMulti-F is used among all scenarios. Figure 8 visualizes the average frequency over all 10 runs of the assignment of masks $\mu ^1$, $\mu ^2$, and $\mu ^3$ to the scenarios of scenario sets $\varLambda ^{52}$ and $\varLambda ^{13}$ for all patient configurations. In general, we confirm that the average use of the masks $\mu ^1$, $\mu ^2$, and $\mu ^3$ is balanced for all patient configurations, so none of the masks is tailored exclusively for one scenario. Instead, we see that each of the three masks is assigned for a cluster of scenarios as targeted in Section 5. For scenario set $\varLambda ^{52}$, we note that mask $\mu ^3$ (which includes the most acute slots) is assigned more often than mask $\mu ^1$ (which includes the fewest acute slots), compared to scenario set $\varLambda ^{13}$. We explain this with the fact that mask $\mu ^1$ based on scenario set $\varLambda ^{52}$ includes less acute slots than mask $\mu ^1$ based on scenario set $\varLambda ^{13}$. At the same time, mask $\mu ^3$ based on scenario set $\varLambda ^{52}$ also includes less acute slots than mask $\mu ^3$ based on scenario set $\varLambda ^{13}$ for patient configurations 1-12. We conclude this section with an example of a mask, a robust mask, and a robust nested multimask resulted from the tenth run of patient configuration 8 as visualized in Fig. 9.

**Fig. 10:**
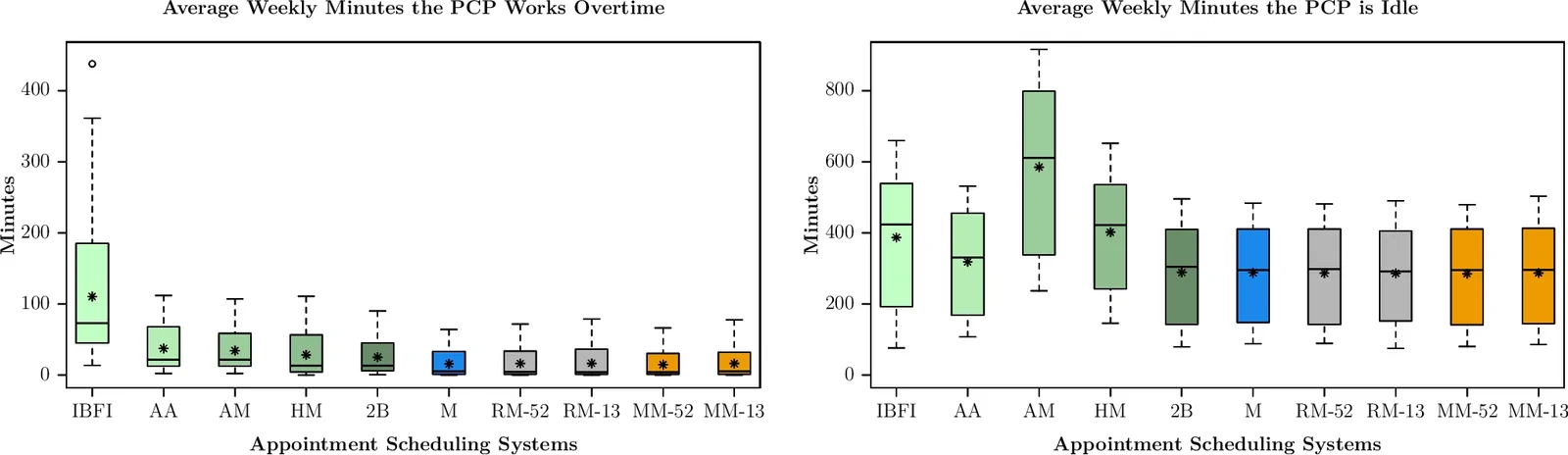
PCP’s average weekly overtime and idle time

### Evaluation by agent-based simulation

In this section, we evaluate the appointment scheduling systems presented in Section 6.1.4. We focus on the comparison of the IBFI and the 2B system with the mask-based systems M, RM, and MM and use the AM, the HM, and the AA system as a benchmark. For the RM and MM appointment scheduling system, we denote whether they are computed on scenario set $\varLambda ^{52}$ or $\varLambda ^{13}$ by RM-52 or RM-13 and MM-52 or MM-13, respectively. In the following, we address RM-52, RM-13, MM-52, and MM-13 as individual appointment scheduling systems. To evaluate the total of ten resulting appointment scheduling systems, we use some of SiM-Care’s key performance indicators. For each individual key performance indicator, we summarize the results of all appointment scheduling systems in Tables 9–18 in Appendix B. In addition, for each individual key performance indicator, we visualize below the results for all appointment scheduling systems of all 180 instances by means of box plots, with the average of these results indicated by a star.

We start with evaluating three key performance indicators for the PCP: the average weekly overtime, the average weekly idle time, and the weekly maximum deviation from the actual to the optimal workload. The results of overtime and idle time are visualized in Fig. 10. We confirm that IBFI causes significantly more weekly overtime than all other appointment scheduling systems. The overtime averages 110.46 minutes. These results are consistent with our expectations as IBFI does not reserve time for potential walk-ins. The remaining appointment systems cause overtime at lower level with averages between 14.61 and 37.45 minutes. In particular, we note that the mask-based systems, which cause between 14.61 and 16.41 minutes of average overtime, perform at least 8.71 minutes better than 2B, the best non-masked-based systems with 25.12 minutes of average overtime. We confirm that MM-52 causes the least amount of average overtime at 14.61 minutes. Overall, we note that the average overtime is relatively low which can be explained by the incorporated buffers and the admission of patients up to a certain utilization threshold.

Considering the idle time, we confirm that AM causes the most average weekly idle time. However, if we consider idle time in relation to overtime, we rate IBFI as the worst performing system. Overall, MM-52 achieves the lowest average weekly idle time in the amount of 285.02 minutes. In fact, all mask-based systems are at least as good as 2B, the non-mask-based system with the lowest average weekly idle time of 288.63 minutes.

**Fig. 11:**
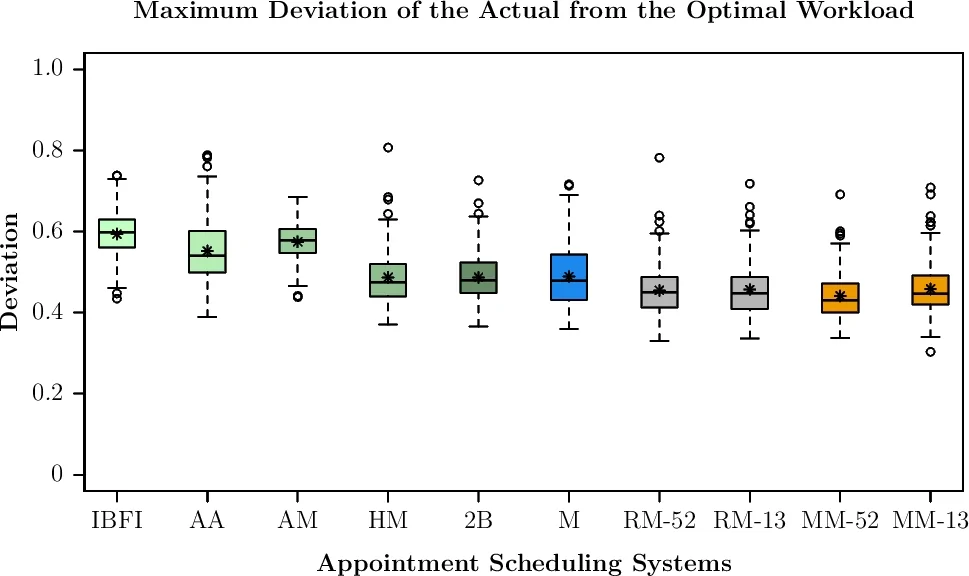
Worst-case deviations of the 52 weeks of all instances, i.e., in each case the worst-case deviation of the actual from the optimal PCP’s workload among all sessions

Finally, across all 52 weeks, we consider the worst-case maximum deviation of the actual from the optimal PCP’s workload among all sessions as visualized in Fig. 11. This corresponds to the definition of the cost of a (robust) mask for the mask-based systems. IBFI causes the largest average worst-case imbalance between the actual and optimal PCP’s workload. This average imbalance is only minimally larger than that caused by AM. Based on the results of overtime and idle time, these results are consistent with our expectations. We further note that HM causes with one instance the maximum worst-case deviation of 0.8072, i.e., the PCP works 0.8072 times the optimal workload more or less in a session. If we consider the non-mask-based systems, we confirm that 2B performs best on average. However, 2B performs on average worse than all mask-based systems except M. Among all appointment scheduling systems, MM-52 causes the smallest average worst-case deviation of 0.4413. In conclusion, compared to the appointment scheduling systems from the literature, the mask-based systems achieve to balance the utilization of the PCP ’s capacity as targeted in Section 3.3.

**Fig. 12:**
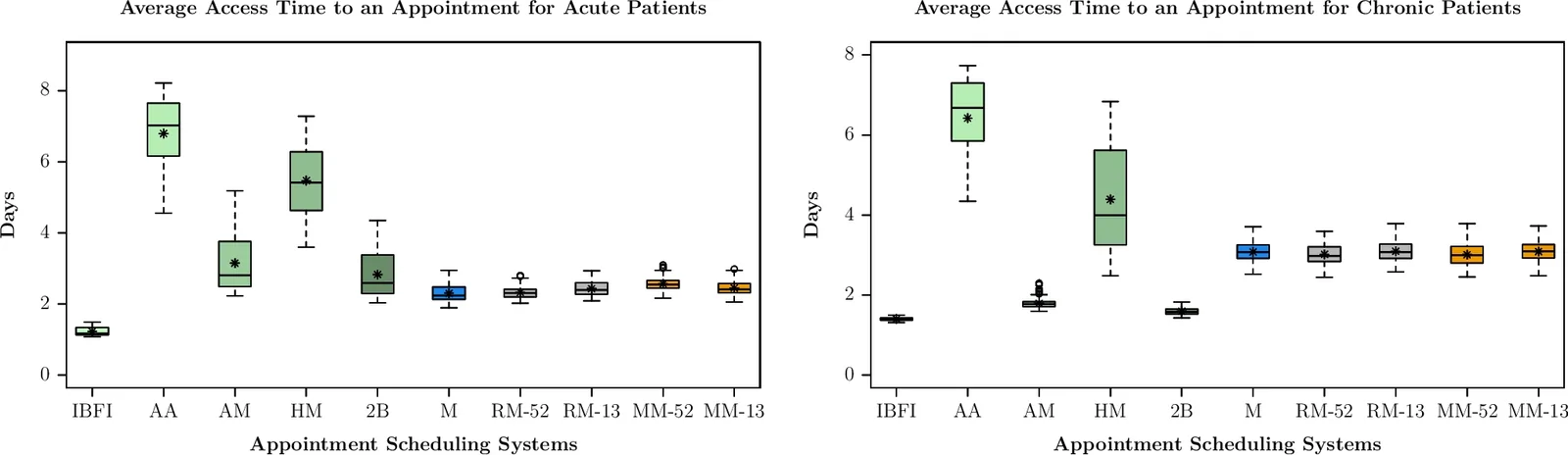
Average Access time to an appointment for acute and chronic patients

In the next step, we consider seven key performance indicators for patients. First, we discuss the average access time to an appointment of acute and chronic patients as visualized in Fig. 12. As expected, IBFI ensures the shortest average access time to an appointment for acute and chronic patients. For instance, for acute patients the maximum and average access times to an appointment are 1.49 and 1.22 days, respectively. Clearly, the benchmark systems AA, AM, HM perform the worst as the slots that allow to accommodate appointments are limited to specific sessions. We confirm that the mask-based systems ensure a faster average access time to an appointment, ranging from 2.30 to 2.57 days, than the 2B system with 2.83 days. However, the average access time to an appointment increases for chronic patients if a mask-based system is used compared to 2B. We explain this by the fact that the non-mask-based systems equally distribute appointment slots for chronic patients among all sessions. If the appointment slots for chronic patients are spread only over a few sessions, as it is the case for the mask-based systems, this may not meet patient preferences and might lead to longer access times. In conclusion, the mask-based systems ensure for all instances a maximum access time to an appointment for acute and chronic patients of three and four days, respectively. This meets the objectives formulated in Section 3.1. At this point, we emphasize that these short access times are guaranteed, although the patients’ willingness to wait in SiM-Care is not limited to three days as assumed in the optimization models. In this study, the average willingness to wait for appointments of acute and chronic patients has been 9.39 and 12.43 days in SiM-Care, respectively. We note that the average willingness to wait of acute patients has been only 6.49 days if the illness ‘vaccination’ with the expected willingness to wait of 40 days (cf. Table 6) is omitted.

**Fig. 13:**
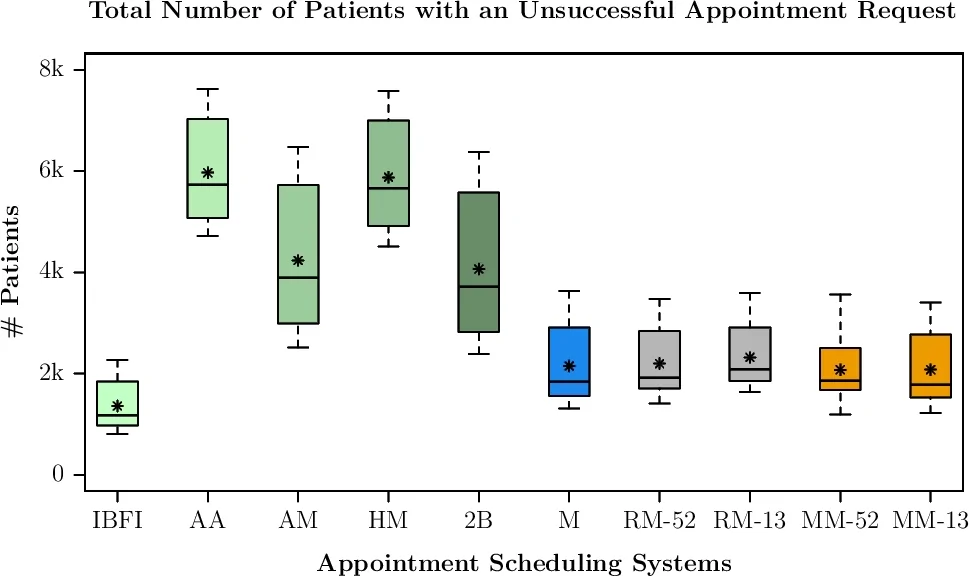
Total number of patients with an unsuccessful appointment request

Second, we consider the total number of unsuccessful appointment requests within a year as visualized in Fig. 13. As IBFI allows all slots to accommodate an appointment, it clearly ensures the smallest number of unsuccessful appointment requests. The results of the benchmark systems AA, AM, and HM confirm that the number of unsuccessful appointment requests increases if appointment slots are restricted to specific sessions or possibly if too few slots exist that allow to accommodate an appointment. We underline that the mask-based systems perform significantly better than 2B. M causes a maximum of 3631 unsuccessful treatment requests which is the maximum number among all mask-based systems and still lower than 4064.61 which is the average number of 2B. Furthermore, MM-52 causes with 2076.05 the smallest average number of unsuccessful treatment requests among all mask-based systems and the second smallest average number among all appointment scheduling systems. Overall, we assess that the number of unsuccessful appointment requests is minimized as targeted in the second optimization stage of the optimization models.

**Fig. 14:**
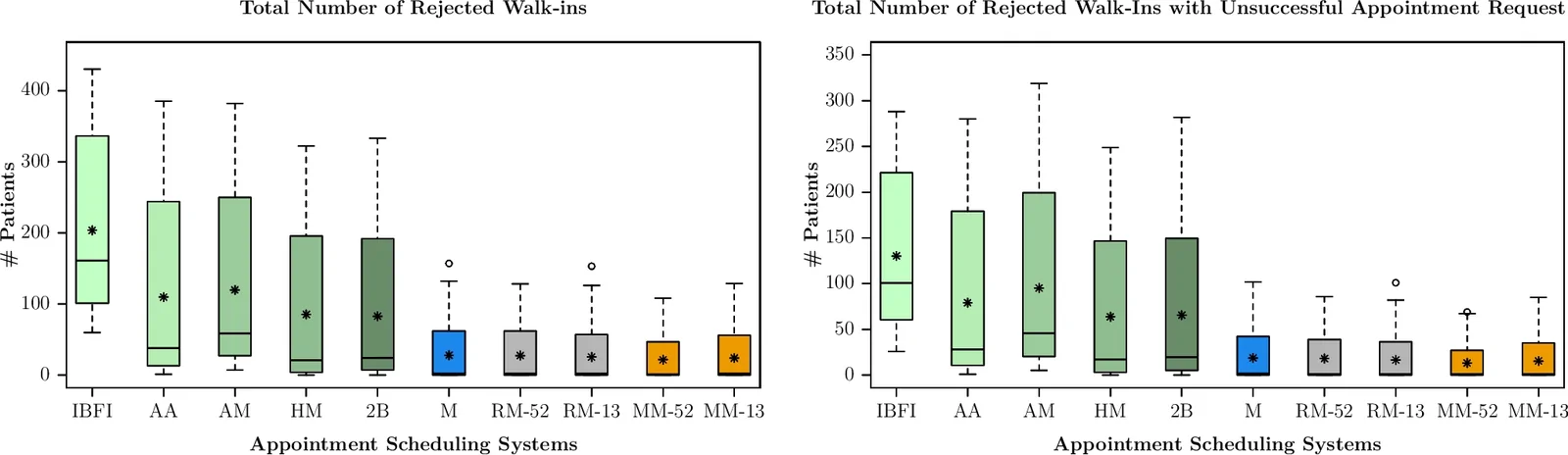
Total number of rejected walk-ins and how many of them previously had an unsuccessful appointment request

**Fig. 15:**
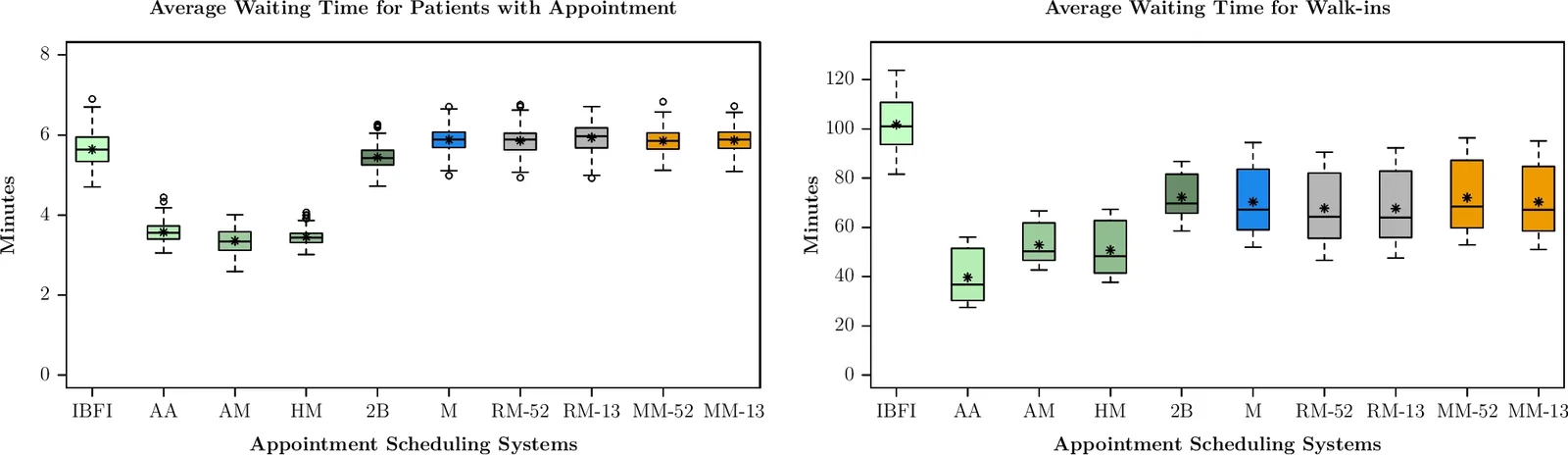
Average waiting time for treatment within the practice of patients with appointment and walk-ins

Third, we consider the total number of rejected walk-ins within a year, i.e., patients that are not seen by the PCP, and how many of them previously had an unsuccessful appointment request. We note that patients with an appointment are always admitted as long as they arrive before the end of the buffer. Furthermore, we note that the admission strategy in SiM-Care does not favor walk-ins who previously had an unsuccessful appointment request over walk-ins who had not requested an appointment. Accordingly, it is random how many patients are rejected twice, once for an appointment request and once for the treatment itself. Nevertheless, it is interesting to evaluate how many patients are rejected twice by the (appointment scheduling) system. The results are visualized in Fig. 14. As IBFI does not reserve slots for potential walk-ins, clearly, it is the system with the highest average total number of rejected walk-ins, namely 203 walk-ins. At the same time, IBFI is the system with the highest average number of rejected walk-ins who previously requested an appointment, even though it is the systems with the fewest average unsuccessful treatment requests. It is also noticeable that the benchmark systems AA, AM, HM and even 2B reject almost three times as many patients on average than the mask-based systems. More precisely, 2B rejects 83 walk-ins on average while the mask-based systems M, RM-52, RM-13, MM-52, and MM-13 reject 28, 27, 25, 22, 24 walk-ins on average, respectively. Furthermore, the maximum and average numbers for the rejected walk-ins who previously had an unsuccessful appointment request are significantly lower for the mask-based systems compared to the non-mask-based systems. Overall, the mask-based systems cause fewer unsuccessful appointment requests, fewer rejected walk-ins, and thus fewer walk-ins who have previously requested an appointment, compared to all other appointment scheduling systems.

Finally, we consider the average waiting times for treatments in the practice for patients with appointments and walk-ins. The results are visualized in Fig. 15. As the treatment strategy is defined so that patients with appointments are always prioritized over walk-ins in the treatment order, waiting times for walk-ins are significantly higher than those from patients with appointments. Considering the waiting times for patients with appointments first, we recall that roughly 20% of patients arrive late for their appointments in SiM-Care. We see that the benchmark systems AA, AM, HM perform the best with an average waiting time ranging between 3.35 and 3.57 minutes. The average waiting times caused by the mask-based systems are comparable to the average waiting time of 5.64 minutes caused by IBFI, they are only a maximum of 0.29 minutes longer. Neglecting the benchmark systems, we confirm that 2B causes with 5.44 minutes the lowest average waiting times for patients with appointments. Considering the waiting times of walk-ins next, we see that the benchmark systems perform again the best with an average waiting time ranging between 39.64 and 52.88 minutes. The mask-based systems cause the next lowest average waiting times for walk-ins ranging between 67.72 and 72.06 minutes. IBFI and 2B provide with 101.76 and 72.26 minutes the worst average waiting time for walk-ins, respectively. We note that MM-52, which causes the maximum waiting time among all mask-based systems, causes still 8.38 minutes less waiting time than the average waiting time caused by IBFI. Furthermore, we note that the maximum waiting time which a walk-in has to wait among all appointment scheduling strategies is caused by IBFI with 123.81 minutes. We conclude this section and refer to the next section where we summarize the results of the mask-based systems and assess them among each other.

## Discussion and conclusion

In this paper, we managed a PCP’s demand for treatment considering three different types of patients by interday appointment scheduling on a tactical decision level. We introduced the MDP and provided a combinatorial interpretation by a network flow and design model. Furthermore, we extended the MDP to the rMDP to obtain a robust setting in which we could account for uncertainties in the weekly distributions of demand. To avoid overfitted solutions and to prevent uncertainties not only in the weekly distribution but also in the total demand, we introduced the rMMDP. For all three problems, we developed a solution approach that combined binary search with compact IP formulations of extension of minimum cost flow problems. We analyzed the optimization models and discussed their results. Finally, we conducted an extensive case study by agent-based simulation in which we evaluated the resulting mask-based appointment scheduling systems and compared them with five adjusted appointment scheduling systems from the literature.

Our study proves on the one hand the potential of the mask-based appointment scheduling systems and on the other hand that they can more than compete with the presented appointment scheduling systems from the literature. The mask-based appointment scheduling systems improve the balanced utilization of the PCP, identifiable by the minimized overtime, idle time, and worst-case deviation of the actual from the optimal workload. Furthermore, they ensure the (timely) access to medical care which includes the following three aspects. First, more patients may receive an appointment so that there are fewer unsuccessful appointment requests. Second, the timely access to an appointment is ensured for acute patients within less than three days and for chronic patients within less than four days. Third, fewer patients are rejected and consequently more patients are admitted for treatment. We point out that the timely access comes at the expenses of waiting times within the practice. To overcome this deficit, future work needs to investigate the order of slots with specific states, especially the positioning of the walk-in slots. Since the walk-in slots are always positioned at the end of a session in the presented appointment scheduling systems, it is not surprising that walk-ins have long waiting times to their treatments. In addition to the positioning of the walk-in slots, walk-ins’ waiting times can be beneficially controlled by an adjusted treatment strategy that does not always prioritize patients with appointments over walk-ins.

Like studies from the literature, our study shows that finding an optimal ratio of scheduled appointments and time for walk-ins in each session is preferable to session that are exclusively for walk-ins. We show that appointments can be used beneficially to distribute the demand for treatment. Compared to studies from the literature, our study achieves remarkable numbers in terms of rejected patients or, in other words, the number of treatments without explicitly targeting this in the objective function. Furthermore, our study provides an appointment scheduling system that is robust against small fluctuations in demand for treatment and additionally adapts to bigger change in demand for treatments.

While this study represents a valuable contribution for interday appointment scheduling in primary care practices, we must not overlook the major limitations of our case study that stem from assumptions and open up directions for further research. The lack of empirical demand data forced us to use a simulation model which can provide rough estimates at best. Furthermore, we could not sufficiently clarify on which scenario set the mask-based appointment scheduling systems work better. The question could be answered depending on available demand data and available time for computation. If the available data allows, future research may also include the analysis of different uncertainty sets. For instance, the rMDP and rMMDP could be adjusted such that a mask is robust within a certain uncertainty budget. A further interesting aspect may be to analyze the influence on the results of variation in the input parameters of the optimization models such as patients’ service times and willingness to wait. We note that the weekly cyclic model must be adjusted to represent a wider range of patients’ willingness to wait. In a cyclic model based on five working days, a willingness to wait of six days would correspond to a willingness to wait of only one day.

Overall, we obtain the following four key findings of this study. First, the distinction in slots reserved for specific patient types is beneficially for the demand management. Second, taking into account information like high walk-in rates in specific sessions has a major impact on the resulting appointment scheduling system and should thus be considered in interday appointment scheduling. Third, a robust nested multimask may be advantageous over a robust mask as observable in key performance indicators such as the number of patients with an unsuccessful appointment request, the number of rejected walk-ins, and the number of rejected patients who previously had an unsuccessful appointment request. We also confirm better results regarding the maximum deviation of the actual from the optimal workload of the PCP. As a result, multiple robust masks that PCPs can switch between allow to respond to changes in demand. On the one hand, PCPs are able to better meet treatment requests of patients, and on the other hand, they achieve a more balanced utilization of their weekly capacity. In future research, however, we need to investigate the circumstances under which the masks of a multimask are activated, i.e., when patients are offered appointments of the second and third mask of the multimask, to fully realize the potential of a multimask. More precisely, we need to analyze and determine at which increase in demand for treatment the next higher mask is activated. Naturally, a multimask can only facilitate the scheduling if the PCP’s capacity matches the demand for treatment. Fourth, our robust and combinatorial network flow and design models are applicable and effective methods to manage demand in primary care practices. More precisely, they are valid modelings for interday appointment scheduling on a tactical decision level. Furthermore, they can be easily adjusted to different settings and withstand a much more complex simulation model on an operational level. We conclude this paper with the confidence that our mask-based appointment scheduling systems meet the effectiveness described in the introduction at the beginning of the paper: The mask-based appointment scheduling systems maintain patient-physician continuity, enable timely access, reduce waiting times, smooth the PCP workflow, and provide robustness against demand uncertainties.

## Figures and Tables

**Figure:**
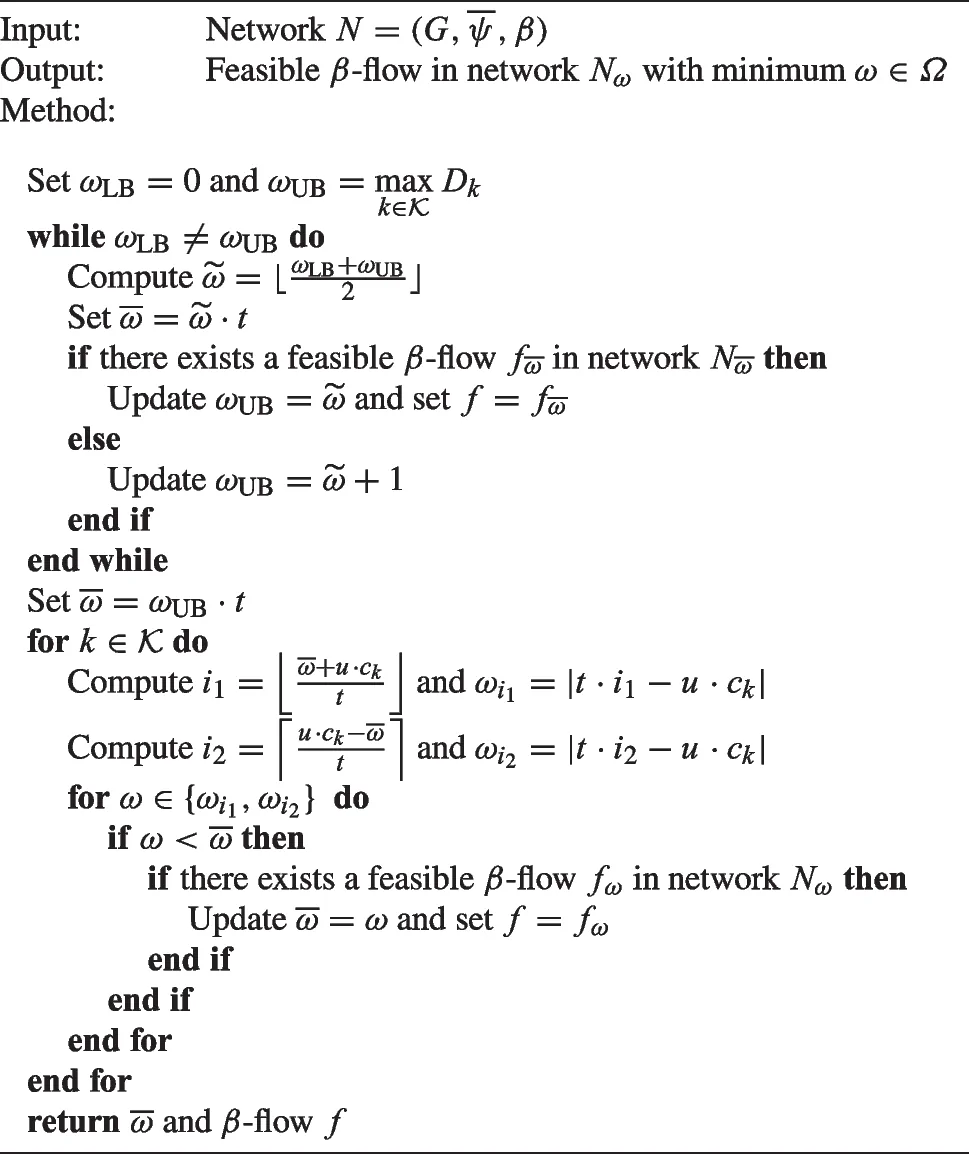
Algorithm 1

**Table 7:**
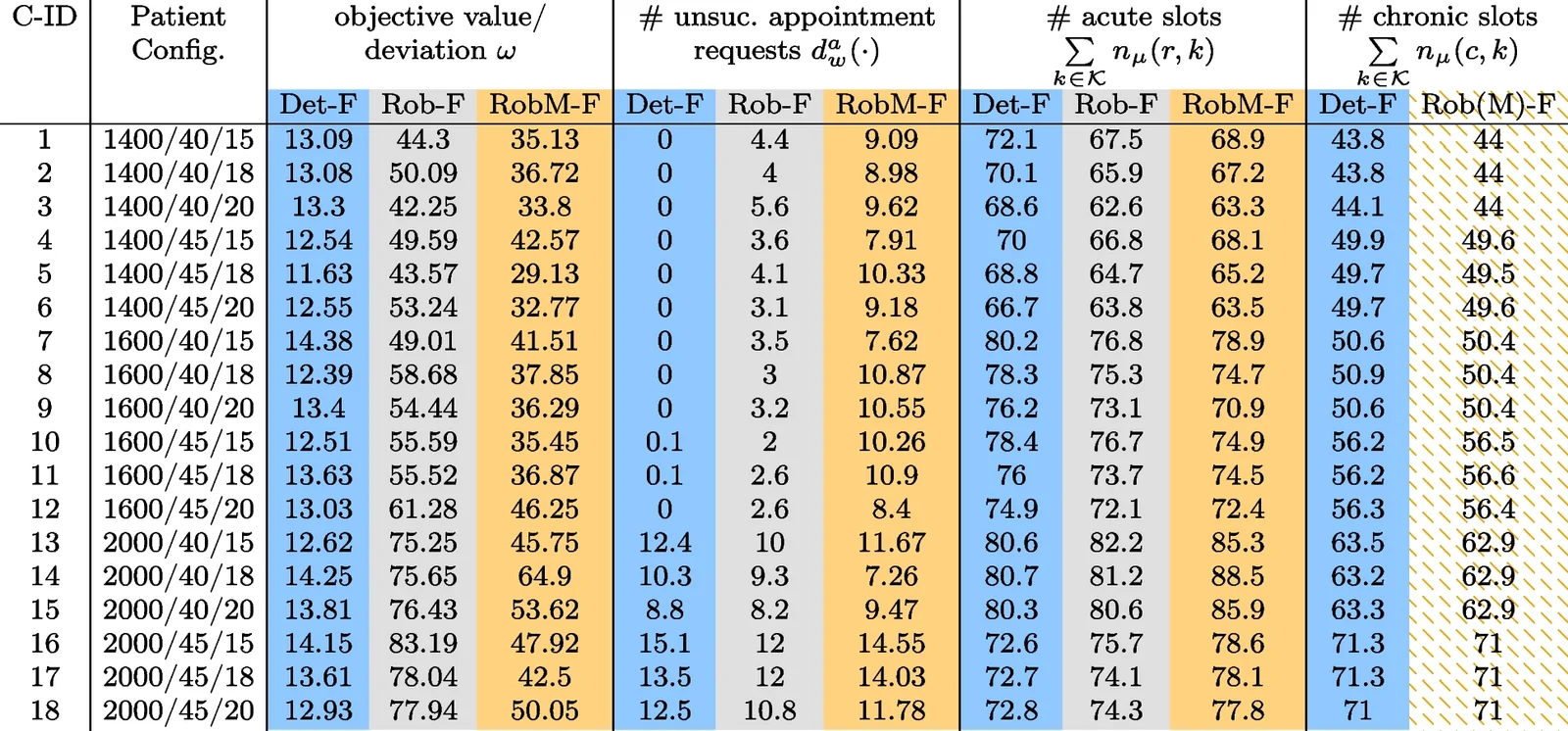
Average computational results of the mask models based on scenario set $\varLambda ^{52}$ for all patient configurations

**Table 8:**
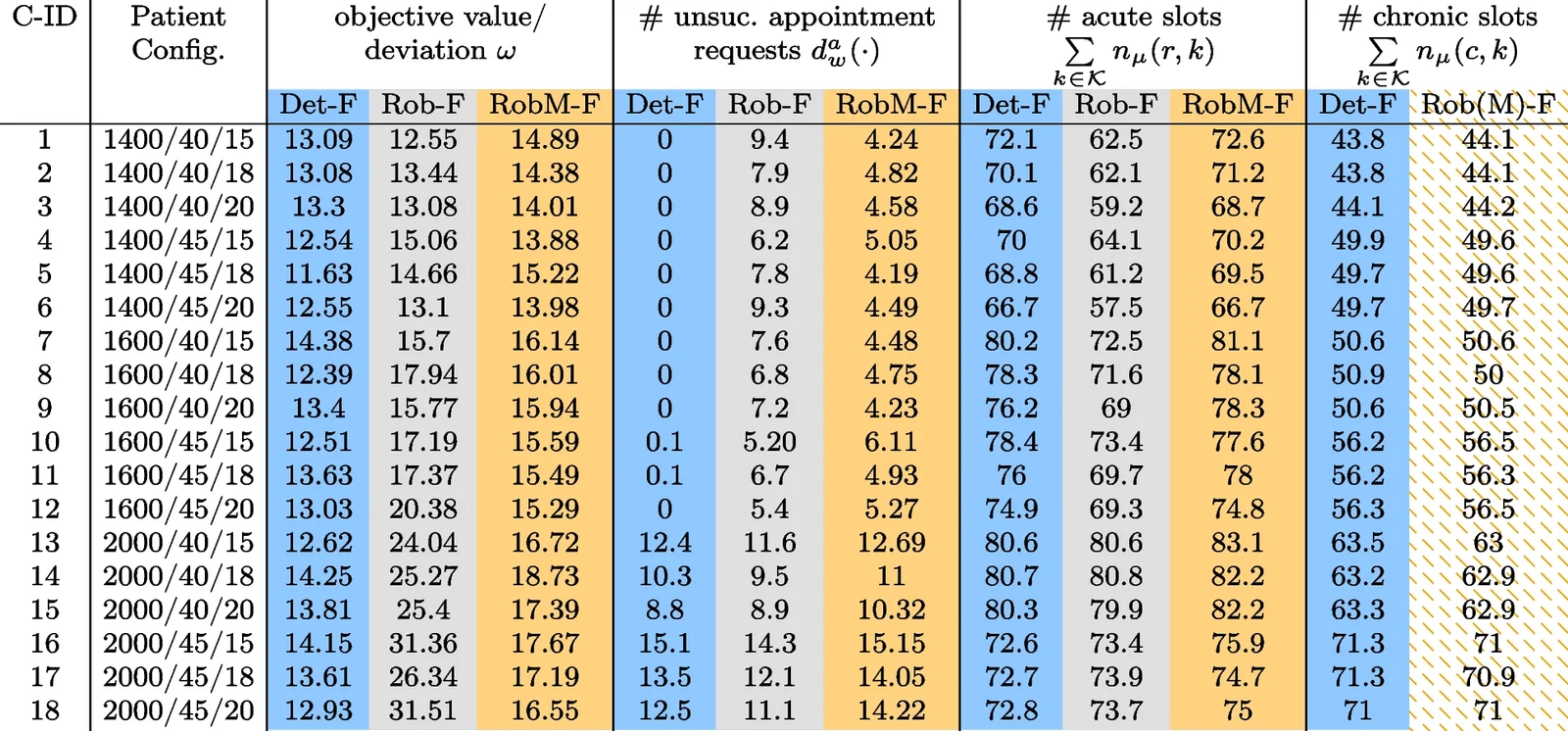
Average computational results of the mask models based on scenario set $\varLambda ^{13}$ for all patient configurations

**Table 9:**
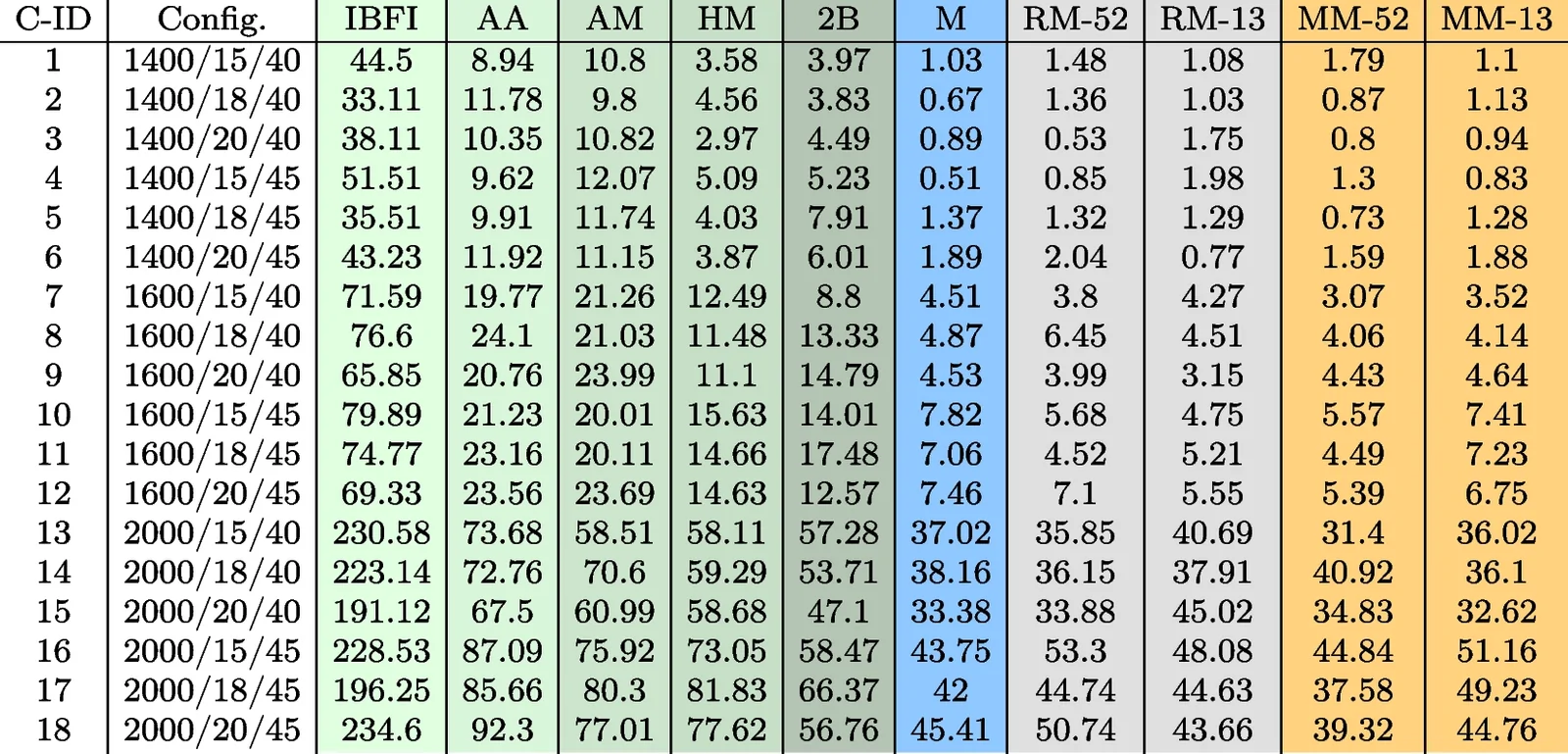
Average overtime of the PCP among the ten runs

**Table 10:**
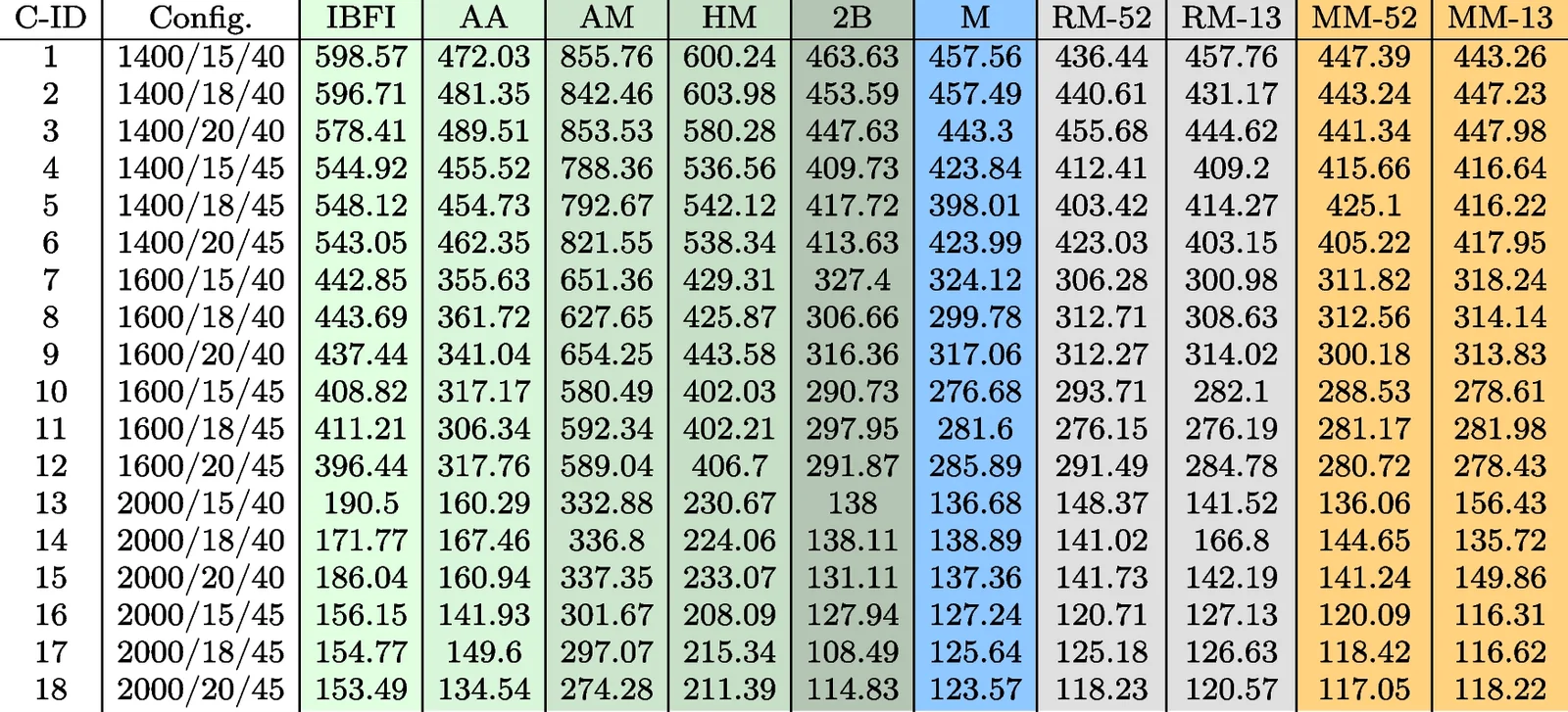
Average idle time of the PCP among the ten runs

**Table 11:**
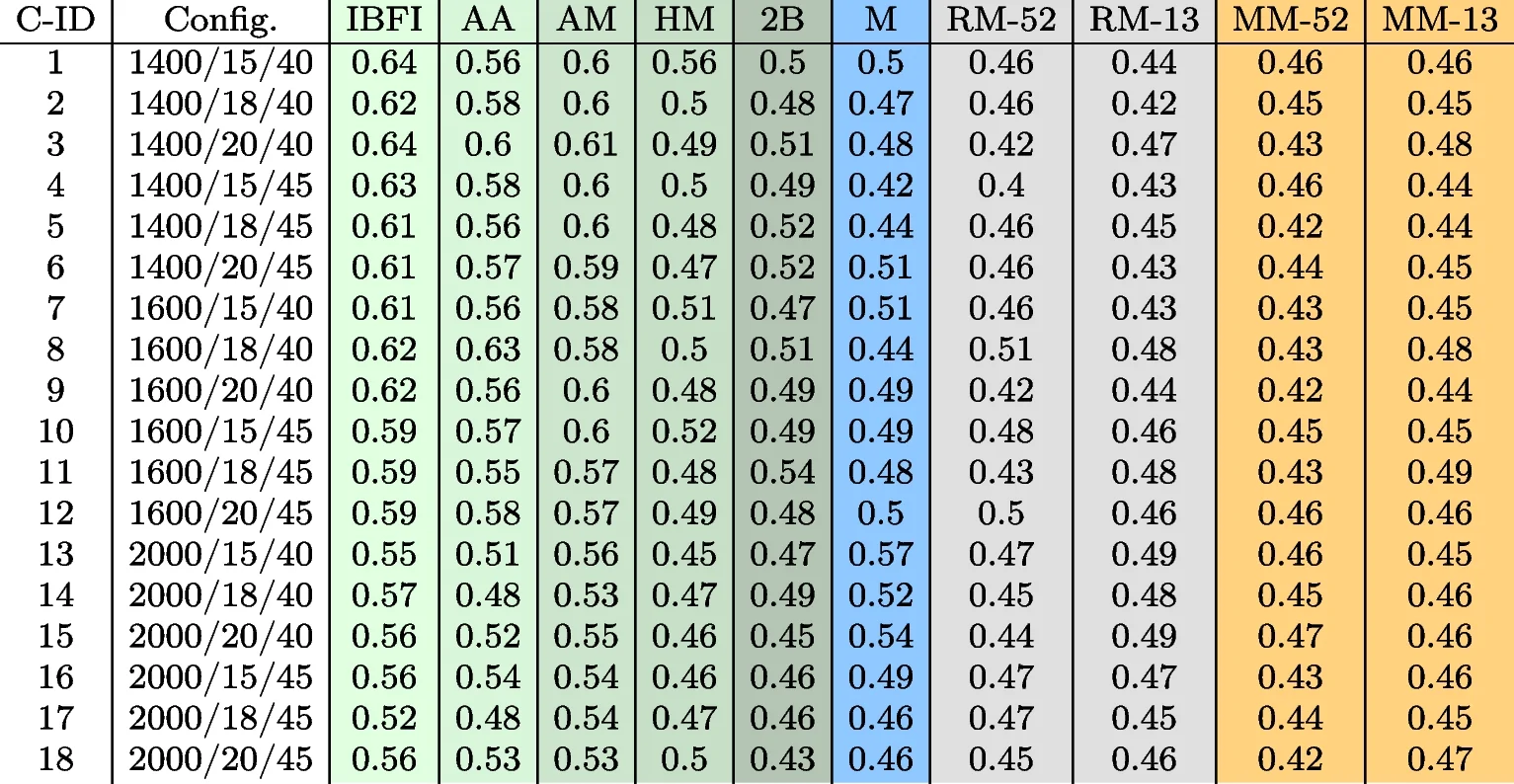
Average maximum deviation of the actual from the optimal PCP’s workload among the ten runs

**Table 12:**
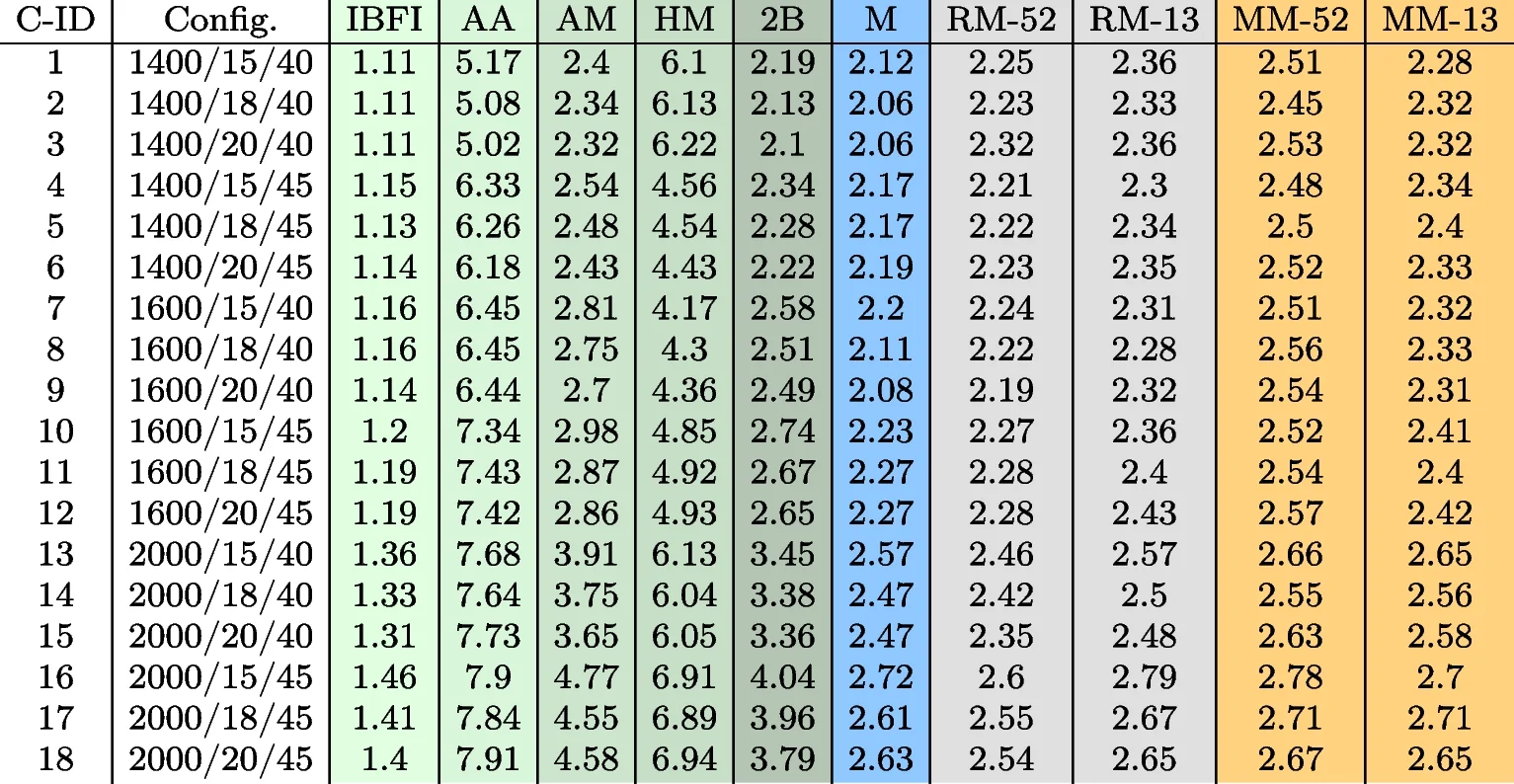
Average access time to an appointment for acute patients among the ten runs

**Table 13:**
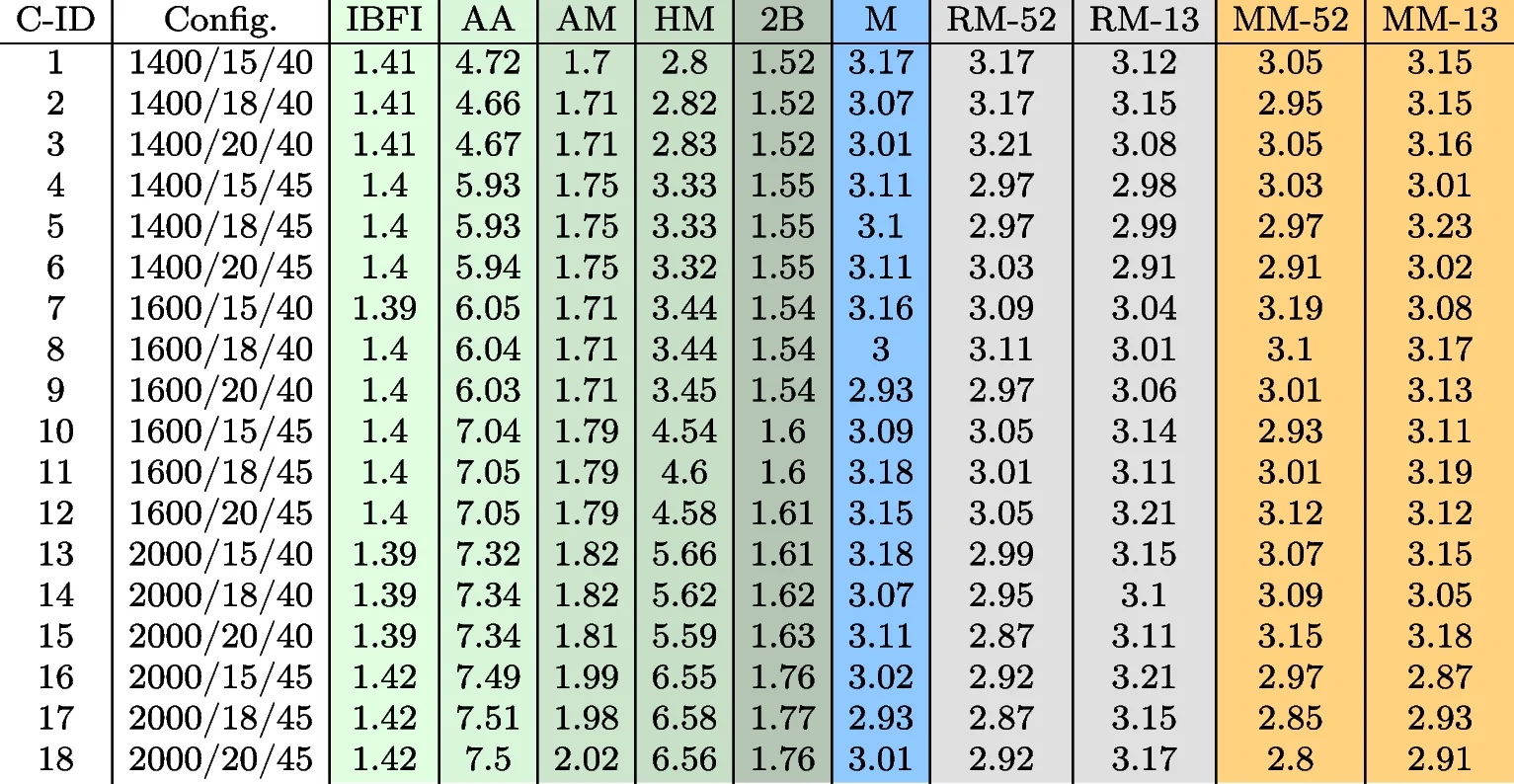
Average access time to an appointment for chronic patients among the ten runs

**Table 14:**
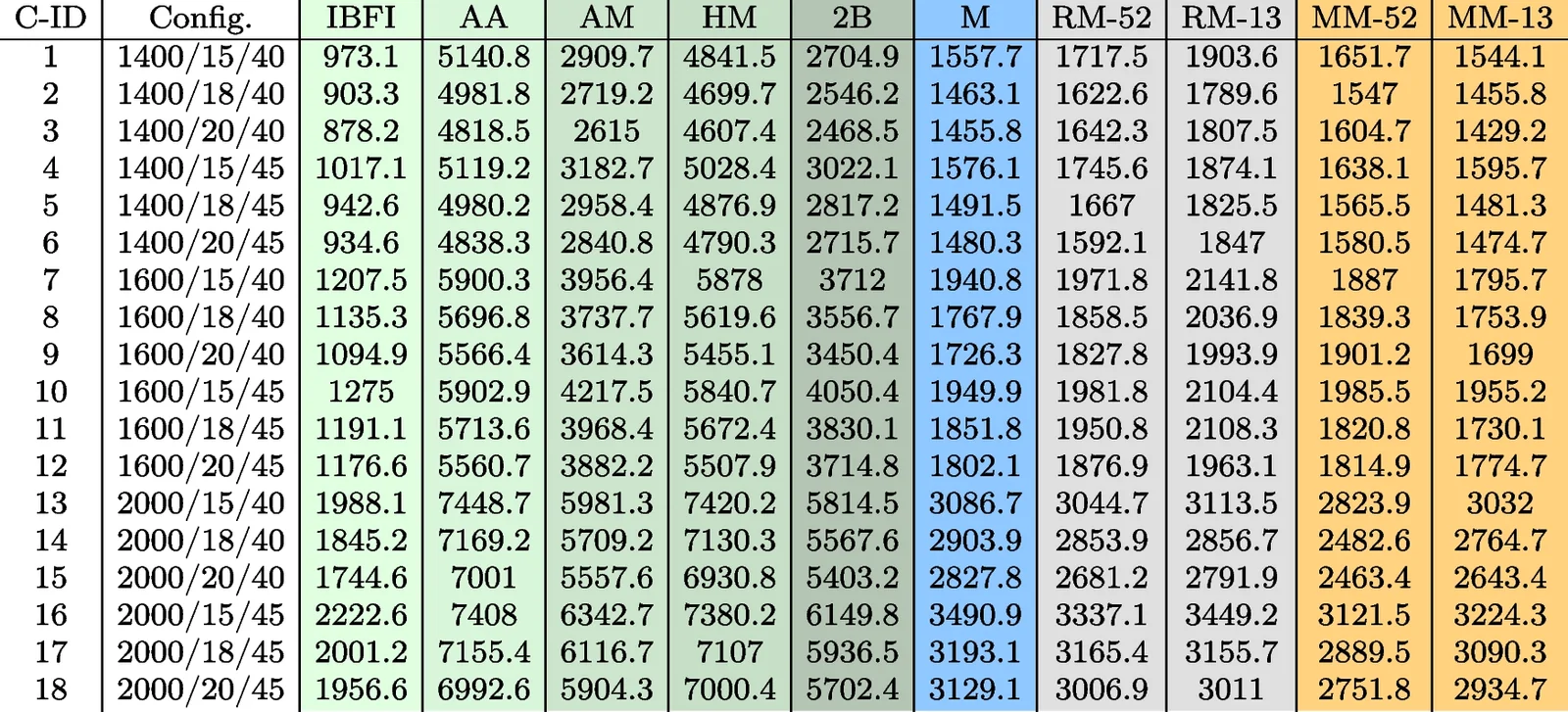
Average total number of patient with an unsuccessful appointment request among the ten runs

**Table 15:**
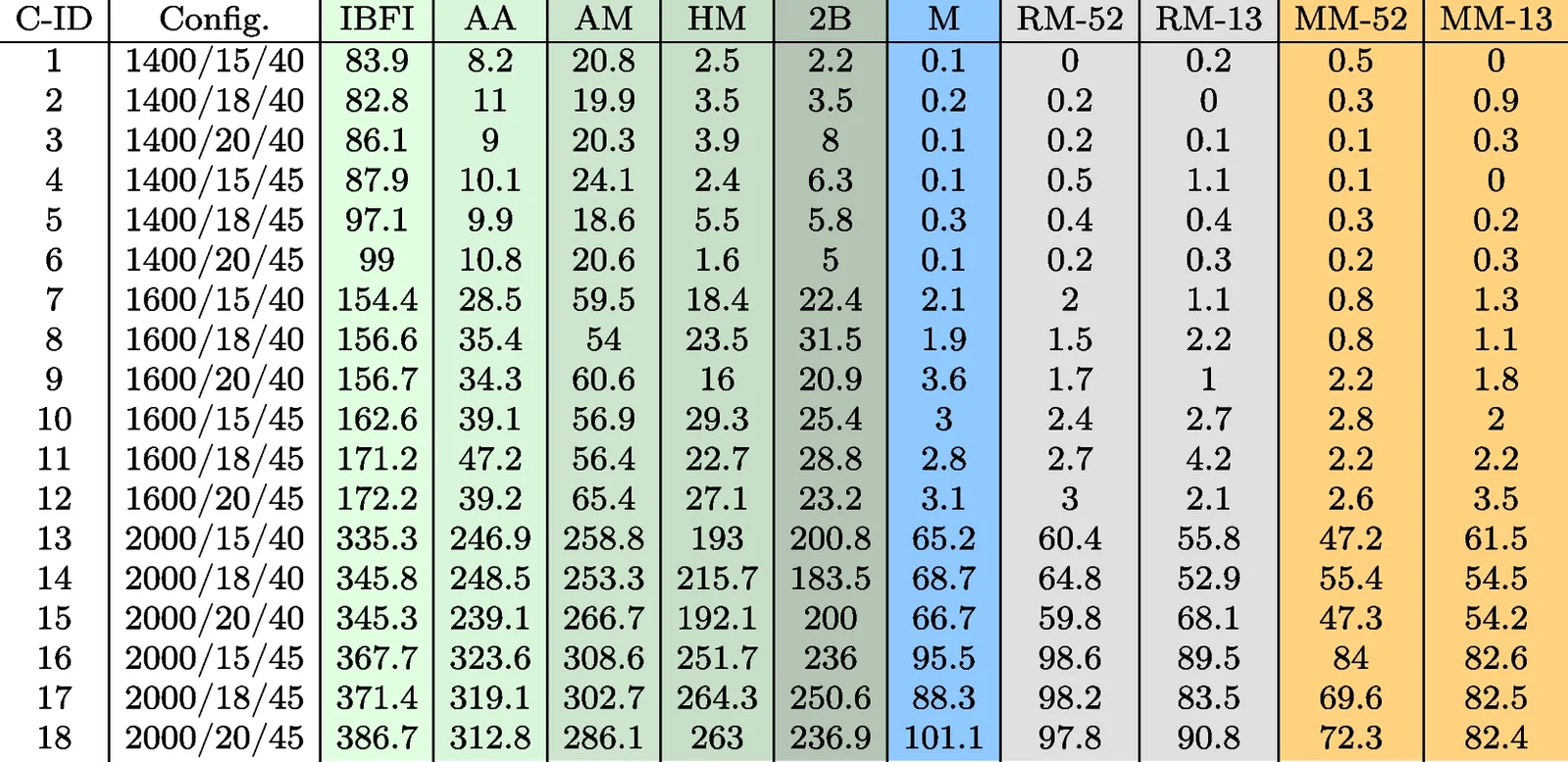
Average total number of rejected walk-ins among the ten runs

**Table 16:**
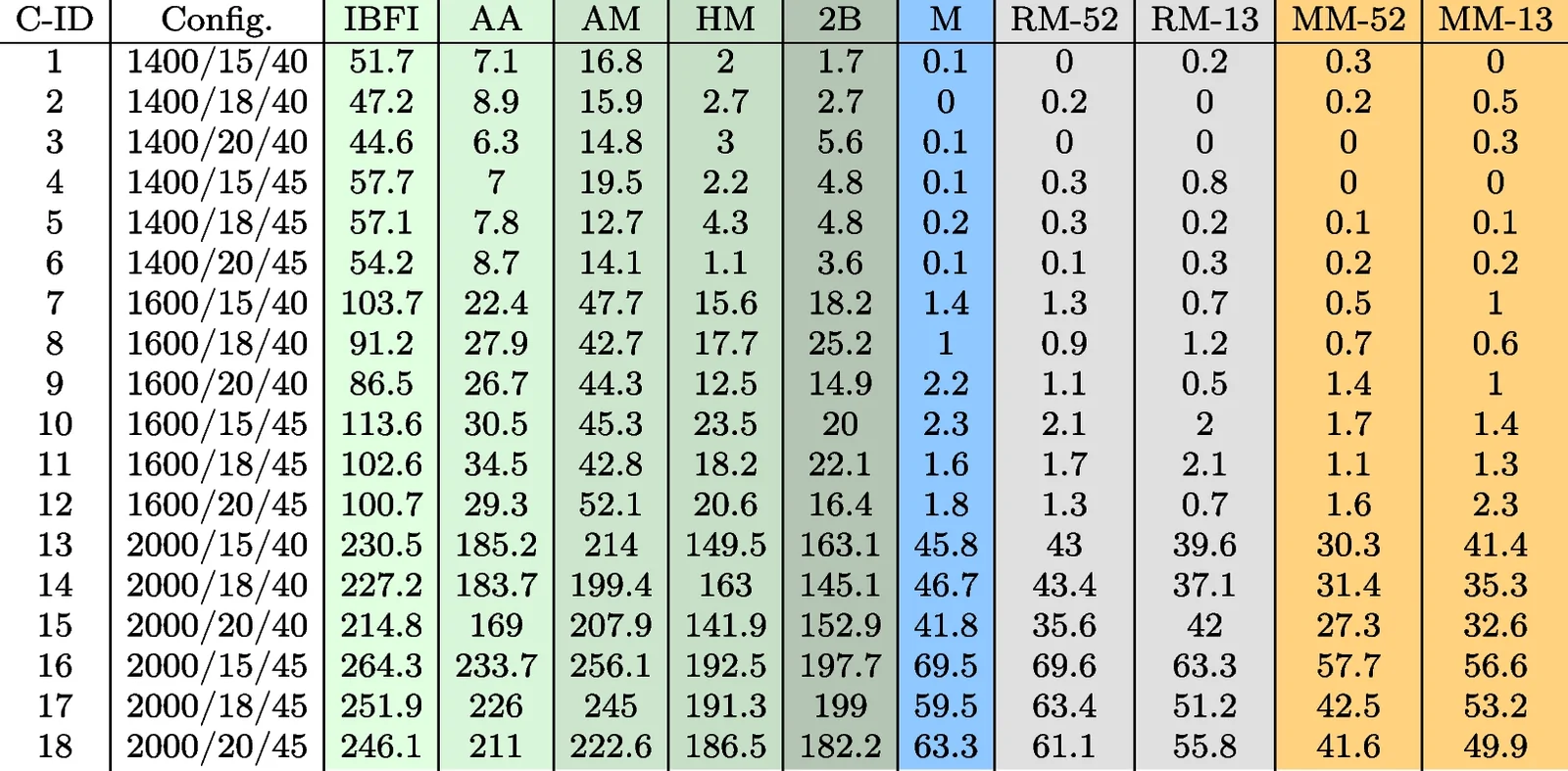
Average total number of rejected walk-ins who previously had an unsuccessful appointment request among the ten runs

**Table 17:**
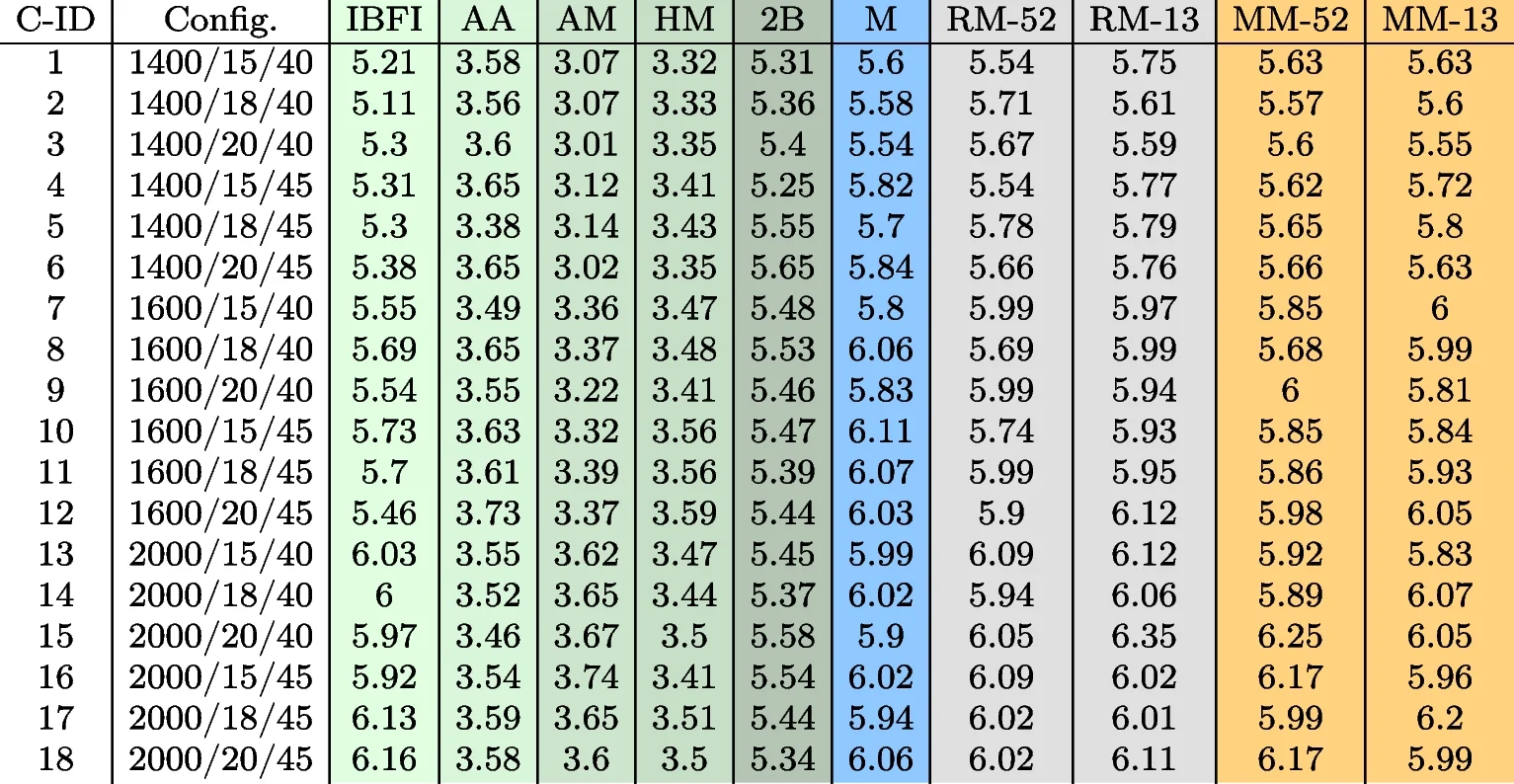
Average waiting time for treatment within the practice for patients with appointments among the ten runs

**Table 18:**
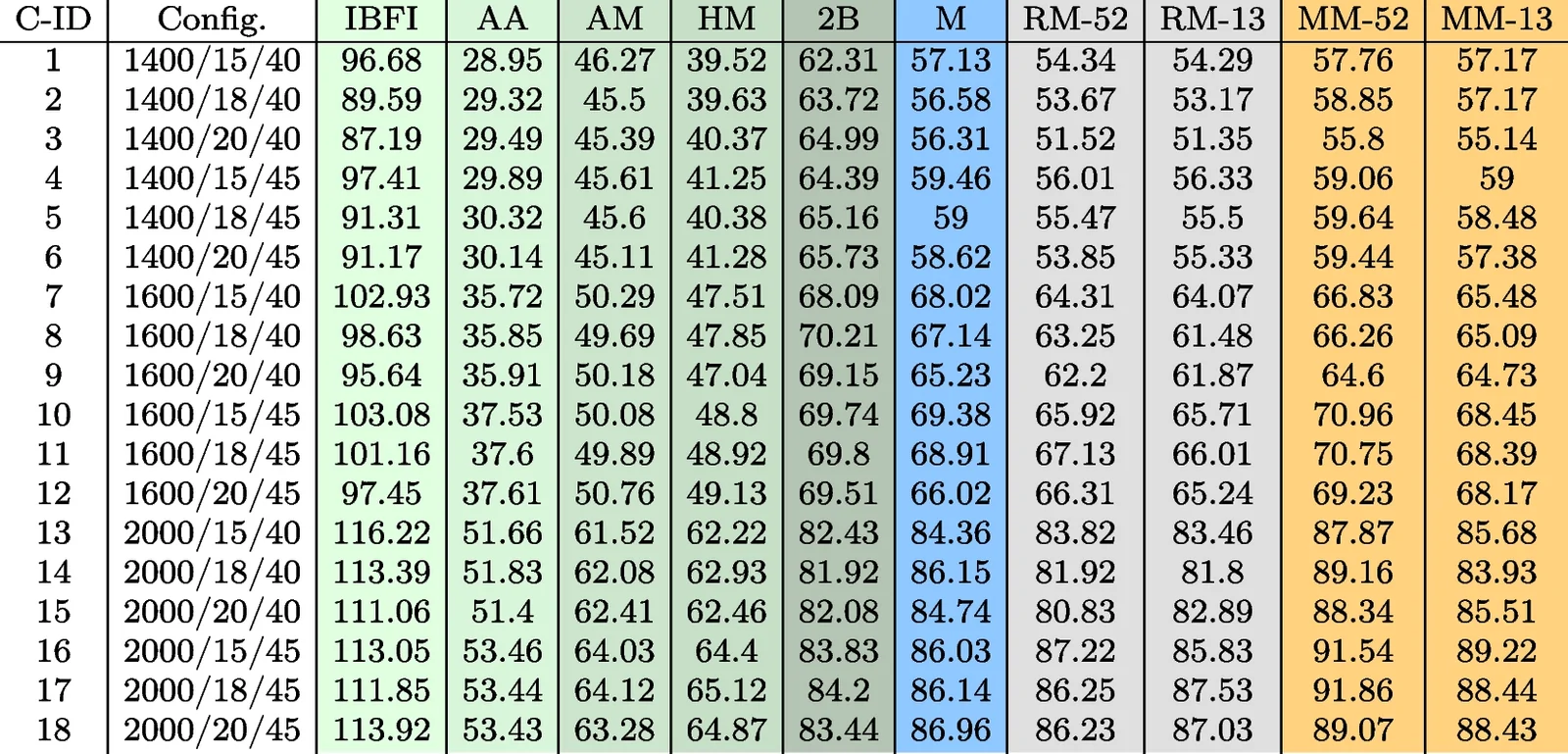
Average waiting time for treatment within the practice for walk-ins among the ten runs

## Appendix A: Algorithm 1

In this section, we present the omitted Algorithm 1 and the proof of Theorem 1.

### Proof (of Theorem 1)

Let $\omega \in \varOmega$ be the parameter and *f* be the β-flow in network $N_\omega$ computed by Algorithm 1. Let $\boldsymbol{\alpha }$ and μ be the corresponding $(d^c,\boldsymbol{d}^a)$-assignment and mask, respectively. Assume there exists a mask $\mu ^{*}$ with less cost, i.e., $c(\mu ^{*})<c(\mu )=\omega$. There exists a $(d^c,\boldsymbol{d}^a)$-assignment $\boldsymbol{\alpha ^{*}}\in \mathcal {A}^{\mu ^*}$ that minimizes the cost of mask $\mu ^*$. Based on assignment $\boldsymbol{\alpha ^{*}}$, we can construct a feasible β-flow $f^*$ in network *N*. We note that each assignment determines a unique flow in network *N*. There exists a parameter $\omega ^*<\omega$ such that $f^*$ is a feasible β-flow in network $N_{\omega ^*}$. This contradicts to the correctness of Algorithm 1.

## Appendix B: Computational results

In this section, we present the computational results of the optimization problems as well as the average results of the evaluation by simulation.
